# A reappraisal of CTLA-4 checkpoint blockade in cancer immunotherapy

**DOI:** 10.1038/s41422-018-0011-0

**Published:** 2018-02-22

**Authors:** Xuexiang Du, Fei Tang, Mingyue Liu, Juanjuan Su, Yan Zhang, Wei Wu, Martin Devenport, Christopher A Lazarski, Peng Zhang, Xu Wang, Peiying Ye, Changyu Wang, Eugene Hwang, Tinghui Zhu, Ting Xu, Pan Zheng, Yang Liu

**Affiliations:** 1 Center for Cancer and Immunology Research, Children’s Research Institute, Children’s National Health System, Washington, DC 20010 USA; 2 OncoImmune, Inc., Rockville, MD 20852 USA; 3 Immutics, Inc., Sunnyvale, CA 94085 USA; 4 Alphamab, Inc., Suzhou, Jiangsu 215125 China

## Abstract

It is assumed that anti-CTLA-4 antibodies cause tumor rejection by blocking negative signaling from B7-CTLA-4 interactions. Surprisingly, at concentrations considerably higher than plasma levels achieved by clinically effective dosing, the anti-CTLA-4 antibody Ipilimumab blocks neither B7 trans-endocytosis by CTLA-4 nor CTLA-4 binding to immobilized or cell-associated B7. Consequently, Ipilimumab does not increase B7 on dendritic cells (DCs) from either *CTLA4* gene humanized (*Ctla4*^*h/h*^) or human CD34^+^ stem cell-reconstituted NSG™ mice. In *Ctla4*^*h/m*^ mice expressing both human and mouse *CTLA4* genes, anti-CTLA-4 antibodies that bind to human but not mouse CTLA-4 efficiently induce Treg depletion and Fc receptor-dependent tumor rejection. The blocking antibody L3D10 is comparable to the non-blocking Ipilimumab in causing tumor rejection. Remarkably, L3D10 progenies that lose blocking activity during humanization remain fully competent in inducing Treg depletion and tumor rejection. Anti-B7 antibodies that effectively block CD4 T cell activation and de novo CD8 T cell priming in lymphoid organs do not negatively affect the immunotherapeutic effect of Ipilimumab. Thus, clinically effective anti-CTLA-4 mAb causes tumor rejection by mechanisms that are independent of checkpoint blockade but dependent on the host Fc receptor. Our data call for a reappraisal of the CTLA-4 checkpoint blockade hypothesis and provide new insights for the next generation of safe and effective anti-CTLA-4 mAbs.

## Introduction

The classic checkpoint blockade hypothesis states that cancer immunity is restrained by two distinct checkpoints: the first is the CTLA-4:B7 interaction that limits priming of naive T cells in lymphoid organs, while the second is the PD-1/B7-H1(PD-L1) interaction that results in exhaustion of effector T cells within the tumor microenvironment.^1^ Since then, several new targets have been under evaluation in clinical trials^2^ and multiple mechanisms have been described for the targeting reagents.^3^ Anti-CTLA-4 monoclonal antibodies (mAbs) induce cancer rejection in mice^4–6^ and patients.^7,8^ Recently, a number of additional mechanisms were proposed to explain the immunotherapeutic effect of anti-CTLA-4 mAbs, including depletion of regulatory T (Treg) cells in tumor microenvironment,^9–11^ and blocking of trans-endocytosis of B7 on dendritic cells (DC).^12,13^ However, it remains to be tested whether the anti-CTLA-4 antibodies induce tumor rejection by mechanisms postulated by the checkpoint blockade hypothesis: namely blocking B7-CTLA-4 interaction and functioning in the lymphoid organs to promote activation of naive T cells.^1^

The systemic effect of anti-CTLA-4 mAbs was questioned by reports proposing that the tumor immunotherapeutic effect of anti-mouse CTLA-4 mAbs depends on their interaction with activating receptor for Fc and that the therapeutic effect correlates with selective depletion of Treg cells in the tumor microenvironment.^9–11^ Although these studies cast doubt on the dogma that anti-CTLA-4 antibodies execute their therapeutic effect at lymphoid organs, they do not address the core issue as to whether blocking the B7-CTLA-4 interaction is required for or contributes to the cancer therapeutic effect, or is involved in the depletion of Treg cells in the tumor microenvironment.

Despite the generally accepted concept that anti-mouse CTLA-4 mAbs induce tumor rejection by blocking negative signaling from the B7-CTLA-4 interaction, the blocking activity of these antibodies^4–6,9–11^ have not been critically evaluated. On the other hand, it has been reported that the clinically used anti-CTLA-4 mAb, Ipilimumab, can block the B7-CTLA-4 interaction if soluble B7-1 and B7-2 were used to interact with immobilized CTLA-4.^14^ However, since B7-1 and B7-2 are membrane-associated co-stimulatory molecules, it is unclear whether the antibody blocks the B7-CTLA-4 interaction under physiologically relevant conditions. Here, we used human *CTLA4* gene knock-in mice as well as mice reconstituted with human hematopoietic stem cells to systematically evaluate whether blocking the B7-CTLA-4 interaction under physiologically relevant conditions is required for the immunotherapeutic effect of anti-human CTLA-4 mAbs. Our data suggest that blocking the B7-CTLA-4 interaction may not contribute to the cancer immunotherapeutic effect. These data have important implications for the development of the next generation of immunotherapeutic anti-CTLA-4 mAbs and call for a reappraisal of the checkpoint blockade hypothesis.

## Results

### Ipilimumab does not block the B7-CTLA-4 interaction if B7 is immobilized or presented on plasma membrane

**Fig. 1:**
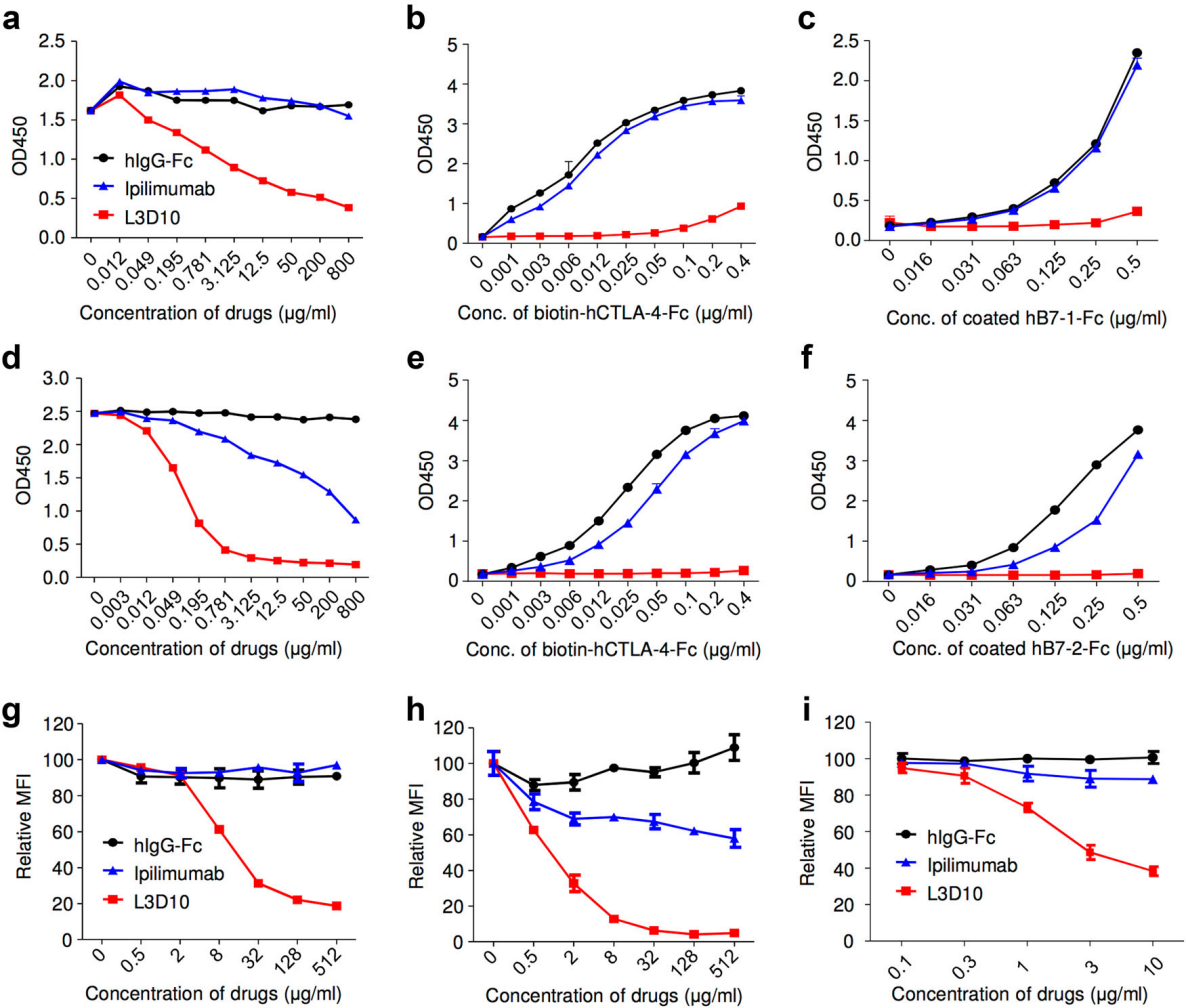
Ipilimumab exhibits poor blocking activity for B7-1-CTLA-4 and B7-2-CTLA-4 interactions if the B7-1 or B7-2 are immobilized. **a**–**c** Blocking activities of anti-human CTLA-4 mAbs Ipilimumab and L3D10 in B7-1-CTLA-4 interaction. **a** hB7-1-Fc was immobilized at the concentration of 0.5 μg/ml. Biotinylated CTLA-4-Fc was added at 0.25 μg/ml along with given doses of antibodies. **b** As in **a**, except that varying doses of biotinylated CTLA-4-Fc was used in the presence of a saturating dose of Ipilimumab or L3D10 (100 μg/ml). **c** As in **a**, except that varying doses of B7-1-Fc was used to coat plate and that a saturating dose of Ipilimumab or L3D10 (100 μg/ml) was used to block CTLA-4-B7-1 interaction. **d**–**f** Blocking activities of anti-human CTLA-4 mAbs Ipilimumab and L3D10 in B7-2-CTLA-4 interaction. **d** As in **a**, except that hB7-2-Fc was immobilized. **e** As in **b**, except that hB7-2-Fc was immobilized. **f** As in **c**, except that hB7-2-Fc was immobilized. Data shown in **a**–**f** are means of duplicate or triplicate optical density at 450 nm. **g** Blocking of CTLA-4 interaction with cell surface hB7-1. CHO cells expressing hB7-1 were incubated with biotinylated CTLA-4-Fc along with given doses of antibodies. The amounts of B7-bound CTLA-4-Fc were detected with PE-streptavidin, and mean fluorescence intensity (MFI) of PE was calculated. **h** Blocking of CTLA-4 interaction with cell surface mB7-2. As in **g**, except CHO cell-expressing mB7-2 was used. **i** Blocking of CTLA-4-Fc binding to spleen DCs matured with overnight LPS stimulation. As in **g** and **h**, except 0.5 μg/ml LPS-stimulated 2 × 10^6^ splenocytes were used for each test and CD11c^high^ DCs (as Fig. 5b) were gated for analyzing PE intensity. Data (mean ± S.D.) shown are normalized MFI values of triplicate samples. Data shown in this figure have been repeated 2–5 times

To facilitate comparative studies, we generated a chimera anti-human CTLA-4 mAb that has the same isotype as Ipilimumab (human IgG1)^14^ using the variable region of a mouse anti-human CTLA-4 mAb (L3D10).^15^ The chimera antibody has an apparent affinity of 2.3 nM, which is similar to Ipilimumab (1.8–4.0 nM).^14,16^ The two antibodies bind to overlapping epitopes on human CTLA-4 in distinct manner based on their binding to mutant CTLA-4 molecules (Supplementary information, Figure S1). Consistent with a previous report,^14^ Ipilimumab potently inhibited the B7-1-CTLA-4 interaction when immobilized CTLA-4 was used to interact with soluble B7-1, which is comparable to L3D10 (Supplementary information, Figure S2A). Since B7-1 and B7-2 function as cell surface co-stimulatory molecules, we evaluated the blockade of anti-CTLA-4 antibodies using immobilized human B7-1 and B7-2. As shown in Fig. 1a, Ipilimumab did not block CTLA-4-Fc binding to hB7-1 immobilized on plates even when used at an extremely high concentration (800 μg/ml). The lack of blocking was not due to batch variation of recombinant Ipilimumab, as we obtained the same results using commercial Ipilimumab from three independent sources (including drug used in the clinic). In contrast, L3D10 showed significant blocking of the binding of hB7-1 immobilized on plates at concentrations as low as 0.2 μg/ml, achieving 50% inhibition (IC_50_) at around 3 μg/ml. Therefore, L3D10 is at least 1000-fold more efficient than Ipilimumab in blocking the B7-1-CTLA-4 interaction when B7-1 is immobilized on the plate. The lack of blocking activity of Ipilimulab was evident across a wide range of ligand and receptor concentrations (Fig. 1b, c). Binding of plate-immobilized B7-2 to CTLA-4-Fc was somewhat more susceptible to blocking by Ipilimumab, albeit at a high IC_50_ of ~200 μg/ml (Fig. 1d). Since the IC_50_ is 10-times higher than the steady plasma levels achieved by the effective dose of 3 mg/kg^7^ (19.4 μg/ml, based on company product inserts), it is unlikely that significant blockade of the B7-2-CTLA-4 interaction would be achieved by the clinical doses. The poor blocking activity of Ipilimumab was observed over a wide range of B7-2 and CTLA-4 protein concentrations (Fig. 1e, f). Again, with an IC_50_ of 0.1 μg/ml, L3D10 is around 2000-fold more efficient than Ipilimumab in blocking the B7-2-CTLA-4 interaction. Perhaps the subtle differences between B7-1 and B7-2 can be explained by the fact that the B7-2-CTLA-4 interaction has a higher off rate^17^ rather than as a result of distinct binding structures because the B7-1-CTLA-4 and the B7-2-CTLA-4 complexes show very similar interactions.^18,19^

To substantiate this surprising observation, we used Chinese Ovary Cells (CHO) that express B7 in conjunction with FcR.^20^ Biotinylated CTLA-4-Fc was used to evaluate the blocking activity of the two anti-human CTLA-4 mAbs. Again, while L3D10 effectively blocked CTLA-4-Fc binding to B7-1-transfected CHO cells, Ipilimumab failed to block even when used at 512 μg/ml (Fig. 1g). Although much less potent than L3D10, high doses of Ipilimumab achieved ~25% blocking of the interaction between human CTLA-4 and mouse B7-1 (mB7-1) (Supplementary information, Figure S2C). While some blocking of CTLA-4-Fc binding to B7-2-transfected and FcR-transfected CHO cells was achieved by Ipilimumab, <50% inhibition was observed even when Ipilimumab was used at 512 μg/ml (Fig. 1h). A potential caveat is that biotinylation may have affected the binding of Ipilimumab to CTLA-4-Fc. To address this concern, we compared binding of L3D10 and Ipilimumab to biotinylated CTLA-4-Fc used in the blocking studies. As shown in Supplementary information, Figure S2B, Ipilimumab is more effective than L3D10 in binding the biotinylated CTLA-4-Fc. Therefore, the failure in blockade by Ipilimumab was not due to insufficient binding to biotinylated CTLA-4-Fc. A similar pattern was observed when polyhistidine-tagged CTLA-4 was used to interact with B7-1-transfected CHO cells (Supplementary information, Figure S2D). To exclude the possible role of FcR on the cell surface, we used hB7-1 expressing and FcR-negative L929 cells. As shown in Supplementary information, Figure S2E, L3D10 but not Ipilimumab, blocked CTLA-4 binding to cell surface B7-1. Furthermore, lack of blocking by Ipilimumab was also observed when LPS-matured spleen DCs were used as the source of B7 (Fig. 1i). Taken together, our data suggest that Ipilimumab’s ability to block B7-CTLA-4 interaction is highly dependent on the assay format employed, with minimal to no detectable blocking activity if B7-1 and B7-2 are immobilized, whereas L3D10 is a robust blocker for the B7-CTLA-4 interaction regardless of whether the B7 protein is immobilized.

**Fig. 2:**
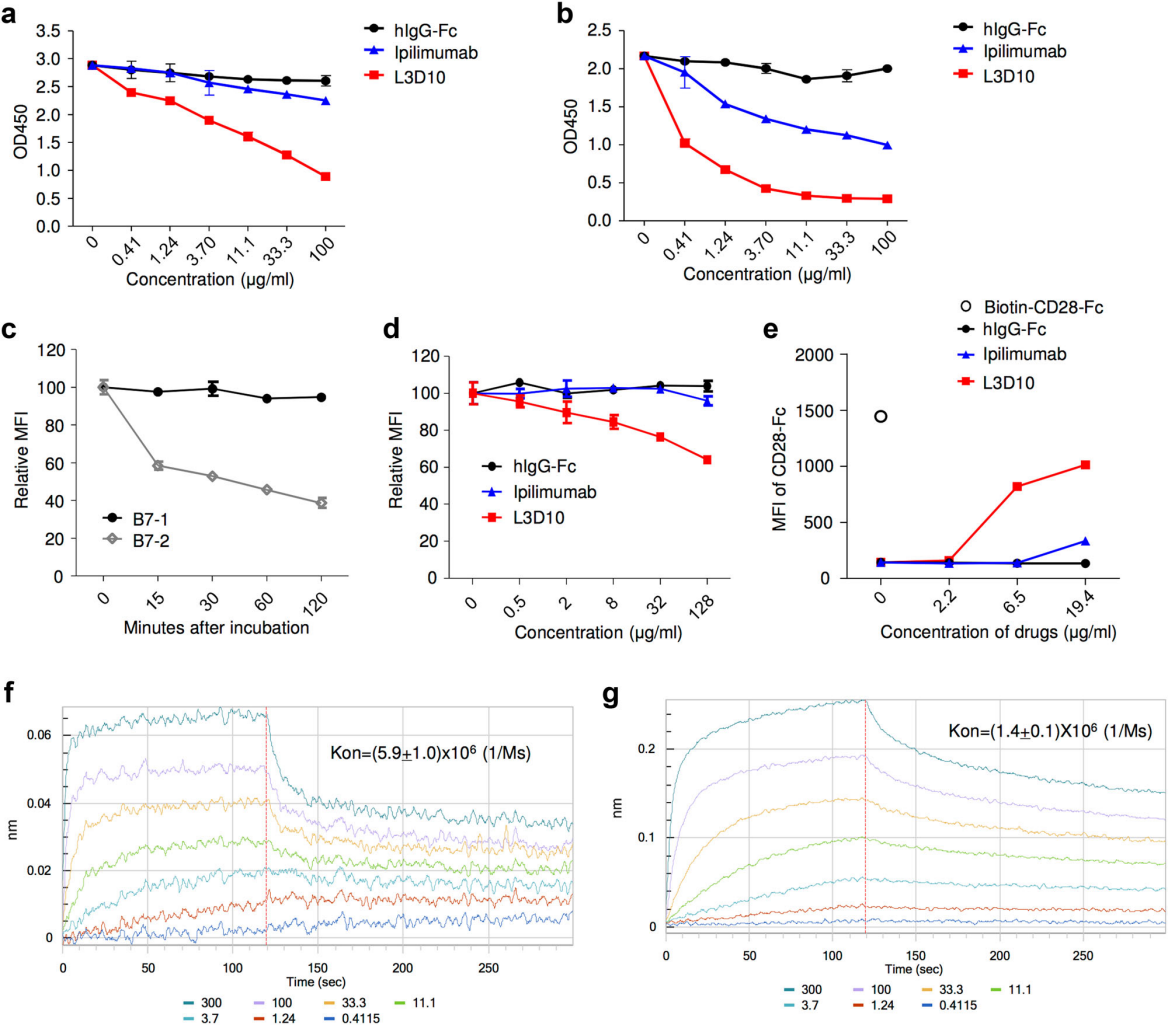
Reconciling the differential blocking effects of Ipilimumab. **a**–**d** Ipilimumab does not break up pre-formed B7-CTLA-4 complex. **a**, **b** Impact of anti-CTLA-4 mAbs on B7-complexed CTLA-4. The B7-CTLA-4 complexes were formed by adding biotinylated CTLA-4 to plates pre-coated with either B7-1 (**a**) or B7-2 (**b**). Graded doses of anti-CTLA-4 mAbs were added to plates with pre-existing B7-1-CTLA-4 complex (**a**) or B7-2-CTLA-4 complex (**b**). After 2 h, the unbound proteins were washed away and the amounts of B7-1 or B7-2-complexed CTLA-4 were detected using HRP-labeled Streptavidin. **c** Dissociation kinetics of B7 and CTLA-4 complex based on flow cytometric assays using B7-expressing CHO cells. Surface hB7-1 or mB7-2-expressing CHO cells (1 × 10^5^/test) were incubated with soluble biotinylated CTLA-4-Fc (200 ng/test) for 30 min at room temperature. After washing, cells were incubated in 100 μl DPBS buffer for the indicated minutes. The amounts of B7-bound CTLA-4-Fc were detected with PE-streptavidin by flow cytometry, and the mean fluorescence intensity (MFI) of PE was calculated from triplicated samples. Data shown are results from one of two independent experiments. **d** L3D10 but not Ipilimumab significantly disrupts the pre-established interaction between soluble CTLA-4 and hB7-1 expressed on CHO cells. Surface hB7-1-expressing CHO cells (1 × 10^5^/test) were incubated with soluble biotinylated CTLA-4-Fc (200 ng/test) for 30 min at room temperature. After washing, cells were incubated with given doses of antibodies in 100 μl DPBS buffer for 1 h. The amounts of B7-bound CTLA-4-Fc were detected with PE-streptavidin, and MFI of PE was calculated. The results represent one of three independent assays with similar patterns. **e** Ipilimumab does not relieve CTLA-4-Fc-mediated inhibition of CD28-Fc binding to B7-1-transfected J558 cells (J558-B7). J558-B7 cells were incubated with biotinylated CD28-Fc (20 μg/ml) in the presence of CTLA-4-Fc (5 μg/ml) and graded doses of anti-CTLA-4 mAbs or control IgG-Fc. Data shown are means and S.E.M. of MFI from triplicate samples and are representative of at least three independent experiments with similar results. **f** Kinetics of B7-1-CTLA-4 interaction when B7-1 was immobilized. **g** Kinetics of B7-1-CTLA-4 interaction when CTLA-4 is immobilized. Data shown in this figure have been repeated two to five times

Since CTLA-4 and B7 co-exist in vivo and interact in a dynamic fashion, efficient blocking would require break-up of pre-existing B7-CTLA-4 complexes. To address this issue, we first allowed B7 to form a complex with biotinylated CTLA-4-Fc. After washing away unbound CTLA-4, graded doses of Ipilimumab or L3D10 were added. After two more hours of incubation, the antibodies and unbound proteins were washed away, and the remaining bound CTLA-4 molecules were detected by HRP-conjugated streptavidin. As shown in Fig. 2a, while L3D10 potently disrupted the pre-existing B7-1-CTLA-4 complex, Ipilimumab failed to do so. Similarly, while high doses of Ipilimumab partially broke down the B7-2-CTLA-4 complex, it was 250-fold less effective than L3D10 (Fig. 2b).

As the first step to evaluate the impact of anti-CTLA-4 antibodies on pre-formed B7-CTLA-4 complex on cell surface, we evaluated the stability of the complex using flow cytometry by incubating the B7-expressing CHO cells with biotinylated CTLA-4-Fc protein at 4 °C for 0–120 min. After washing away any disassociated CTLA-4-Fc, we used PE-conjugated Streptavidin to measure cell-bound CTLA-4-Fc. As shown in Fig. 2c, the amounts of CTLA-4-Fc on B7-1-expressing CHO cells remained unchanged throughout the 120 min of study duration, thus allowing us to test the impact of anti-CTLA-4 antibodies on disrupting the pre-formed B7-1-CTLA-4 complex. In contrast, the B7-2-CTLA-4-Fc complex rapidly dissociated within 15 min, with majority of the complex collapsing within 30 min (Fig. 2c). This rapid disassociation made it impossible to evaluate the impact of anti-CTLA-4 mAbs on pre-formed B7-2-CTLA-4 complex in our assays. As shown in Fig. 2d, Ipilimumab was minimally effective in disrupting the pre-formed B7-1-CTLA-4 complex on cell surface.

Since CTLA-4 has a higher affinity for B7 than CD28-Fc,^17,21^ blocking CTLA-4 may relieve its inhibition of CD28-B7 interaction. To test if L3D10 and Ipilimumab can reverse this inhibition, we added graded amounts of each antibody or control IgG-Fc along with biotinylated CD28-Fc and unlabeled CTLA-4-Fc, and measured the binding of CD28-Fc to B7-1-transfected J558 cells.^22^ As shown in Fig. 2e, L3D10 but not Ipilimumab, significantly rescued the B7-CD28 interaction. The inability of Ipilimumab to break the pre-formed complex suggests that the kinetics of the B7-CTLA-4 interaction will be a key determinant of the blocking activity of Ipilimumab. We therefore evaluated the kinetics of the B7-CTLA-4 interaction by using either immobilized B7-1 or CTLA-4. When B7-1 is immobilized, the apparent affinity of bivalent B7-1-Fc and CTLA-4-Fc is 9.9 × 10^−10^ M (Fig. 2f), which is a somewhat higher value than when CTLA-4-Fc is immobilized (1.5 × 10^−9^ M) (Fig. 2g). Remarkably, the on-rate of CTLA-4 to immobilized B7-1, *K*_on_ = 5.9 × 10^6^ (1/Ms) (Fig. 2f), is four times higher than that of B7-1 to immobilized CTLA-4, which is 1.4 × 10^6^ (1/Ms) (Fig. 2g) (*P* = 0.0015). The slower formation of the B7-CTLA-4 complex when B7 is present in solution may allow Ipilimumab to occupy CTLA-4 prior to formation of the B7-CTLA-4 complex which is resistant to breakup by Ipilimumab, thus providing a mechanism to reconcile the assay-dependent blocking activity of Ipilimumab. On the other hand, L3D10 can break up pre-formed complex, and can thus block the CTLA-4-B7 interaction regardless of the conditions employed.

### Ipilimumab does not effectively block B7-CTLA-4-mediated cell–cell interaction and trans-endocytosis of B7-1 and B7-2 by CTLA-4

**Fig. 3:**
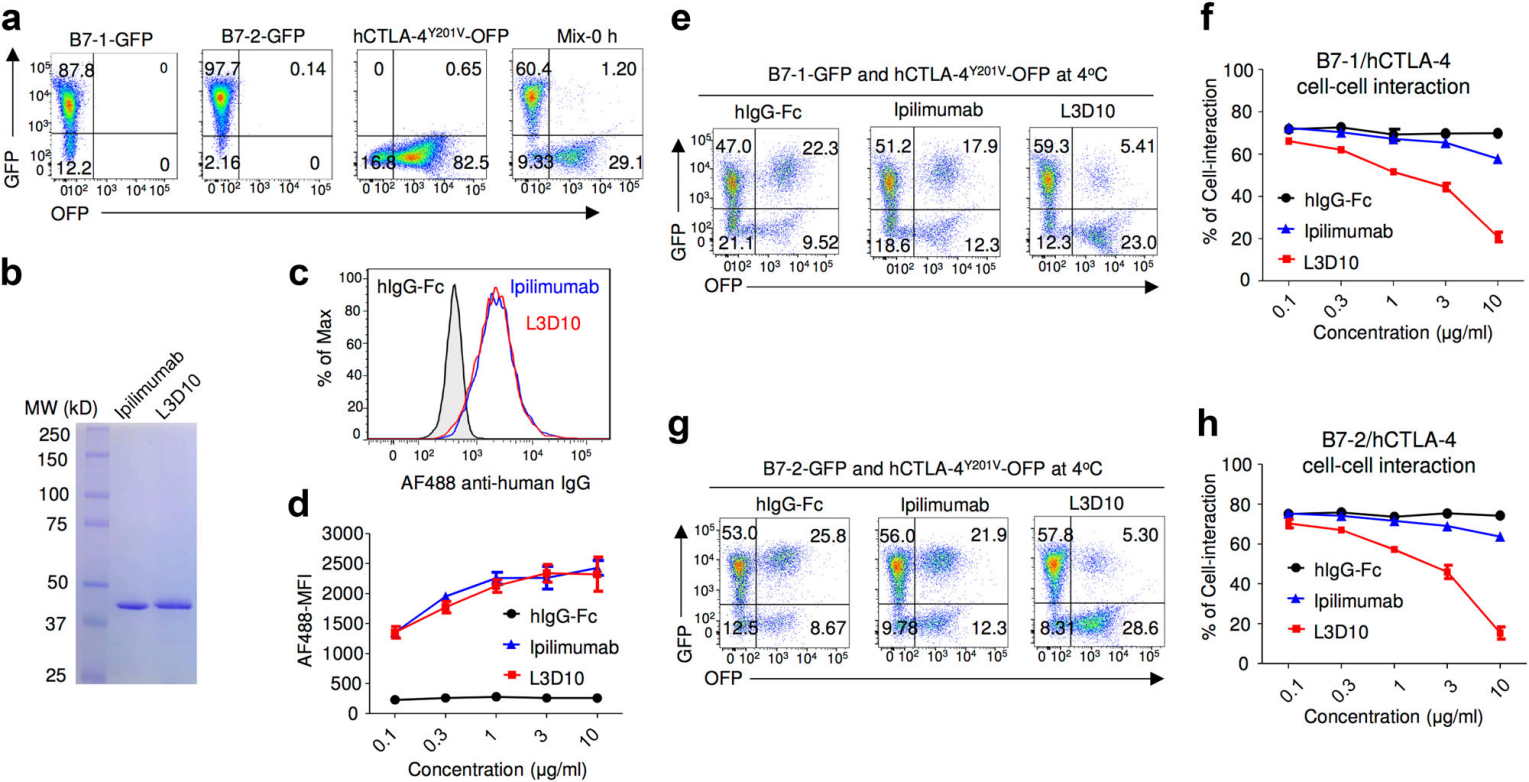
Ipilimumab is ineffective in blocking B7/CTLA-4-mediated cell–cell interactions. **a** Profiles of B7-1-GFP or B7-2-GFP-transfected CHO cells or CTLA-4^Y201V^-transfected 293T cells or mixture of B7-2 and CTLA-4 transfectants without co-incubation. **b** SDS-PAGE analysis for purify of Fabs used for the study. **c**, **d** Representative FACS profiles (**c**, Fabs used at 10 μg/ml) and dose responses (**d**) showing comparable binding by L3D10 and Ipilimumab Fabs to CTLA-4-OFP-transfected CHO cells. Alex Fluor 488-conjugated goat anti-human IgG (H+L) was used as the secondary antibody for the binding assay. Dose responses show similar binding activity of Ipilimumab and L3D10 Fabs. AF488-MFI, mean fluorescence intensity of Alex Fluor 488 dye. **e** Inhibition of B7-1-CTLA-4^Y201V^-mediated cell–cell interaction by anti-CTLA-4 mAb Fabs. B7-1-GFP-transfected CHO cells and CTLA-4^Y201V^-transfected 293T cells were co-incubated at 4 °C for 2 h in the presence of 10 μg/ml Fab or control proteins. Data shown are representative FACS profiles. **f** Quantitative comparison between L3D10 and Ipilimumab for their blocking of cell–cell interaction mediated by B7-1 and CTLA-4 expressed on opposing cells. As in **e**, except that graded doses of antibodies were added. **g** Inhibition of B7-2-CTLA-4^Y201V^-mediated cell–cell interaction by anti-CTLA-4 mAb Fabs. As in **e**, except that B7-2-GFP transfectants were used. **h** Quantitative comparison between L3D10 and Ipilimumab for their blocking of cell–cell interaction mediated by B7-2 and CTLA-4 expressed on opposing cells. As in **f**, except that B7-2-GFP-transfected CHO cells were used. All assays have been repeated at least two times

Most CTLA-4 molecules reside inside the cells as a result of an AP-2-mediated mechanism.^23,24^ In order to measure whether anti-CTLA-4 mAb could block the B7-CTLA-4 interaction when they are both stably expressed on the cell surface, we introduced the Y201V mutation into CTLA-4 to abrogate its spontaneous endocytosis and thus allow stable cell surface expression^25^ (Supplementary information, Figure S3A). As shown in Fig. 3a, CHO cells expressing either B7-1-GFP or B7-2-GFP and HEK293T cells expressing CTLA-4^Y201V^-OFP are clearly distinguishable by flow cytometry. When they were mixed immediately prior to FACS analyses, barely any GFP^+^OFP^+^ cells were observed. To compare Ipilimumab and L3D10 for their ability to block cell–cell interactions, we prepared Fab fragments from both antibodies (Fig. 3b) in order to avoid indirect effects caused by cross-linking of CTLA-4 molecules. The antibody Fabs showed comparable binding to cells stably transfected with OFP-tagged CTLA-4 (Fig. 3c, d). After 2 h of co-incubation at 4 °C without the blocking antibody, most of the OFP^+^ cells acquired GFP at equal intensity to the B7-GFP-expressing cells (Fig. 3e, g). Notably, the GFP^+^OFP^+^ cells had forward and side scatters consistent with cell clusters (Supplementary information, Figure S3B). As shown in Fig. 3e, f, effective blocking of the B7-1-GFP-CTLA-4^Y201V^ interaction was achieved by L3D10 but not Ipilimumab Fab. Similarly, while only 15% inhibition of cellular B7-2 and CTLA-4 interaction was achieved by 10 μg/ml of Ipilimumab Fab, the same dose of L3D10 Fab caused 80% inhibition (Fig. 3g, h).

**Fig. 4:**
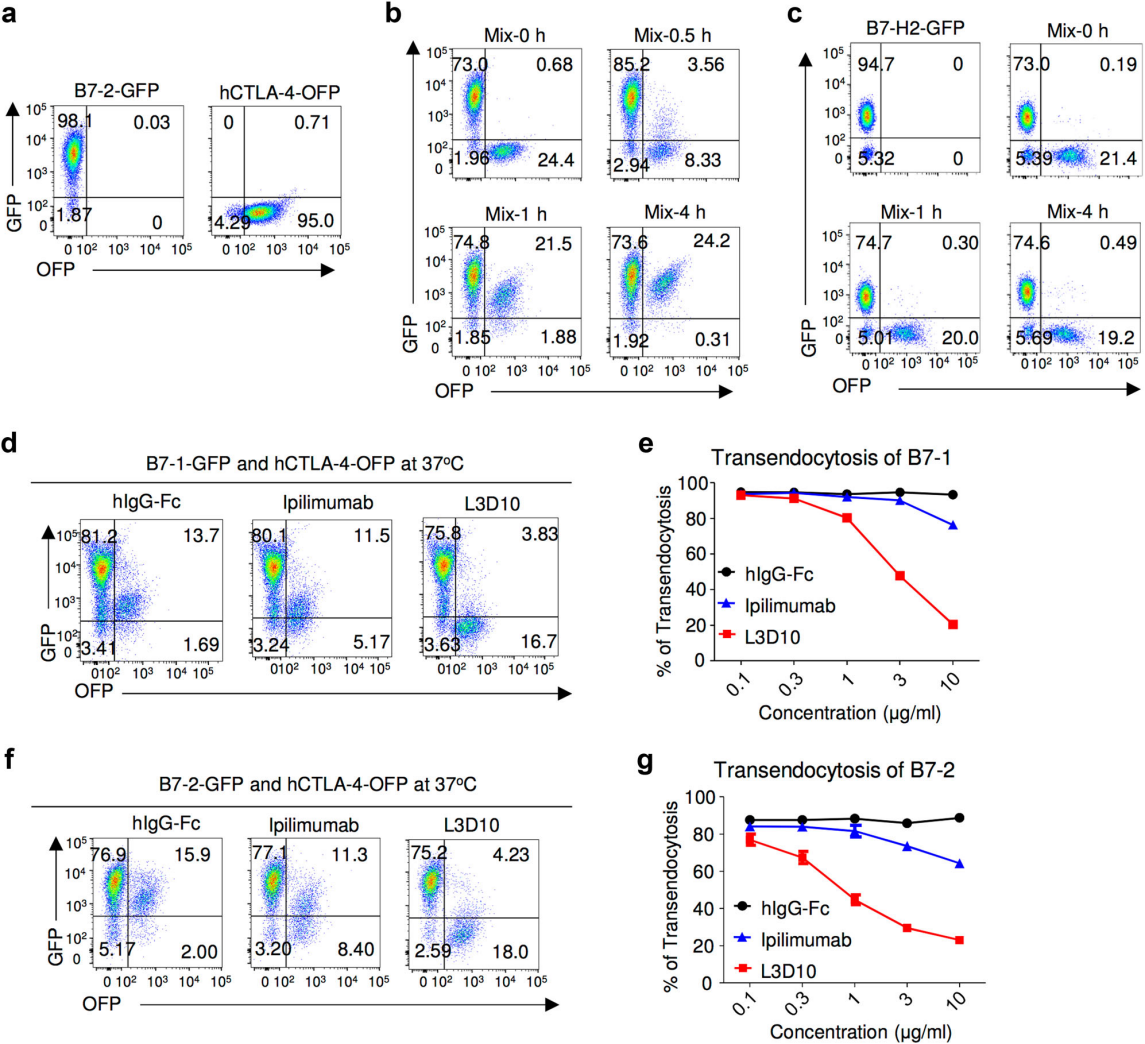
Ipilimumab is ineffective in blocking B7-trans-endocytosis by CTLA-4. **a** FACS profiles of B7-2-GFP-transfected or CTLA-4-OFP-transfected CHO cell lines used for trans-endocytodosis assay. **b** Rapid trans-endocytosis of B7-2 by CTLA-4. B7-2-GFP transfectants and CTLA-4-OFP transfectants were co-incubated for 0, 0.5, 1 and 4 h at 37 °C. **c** Lack of trans-endocytosis of B7-H2 by CTLA-4. As in **b**, except that B7-H2-GFP-transfected P815 cells and data at 0, 1 and 4 h of co-culturing are presented. **d** Representative profiles depicting differential blockade of trans-endocytosis of B7-1-GFP by CTLA-4-OFP-expressing CHO cells during coculture in the presence of control hIgG-Fc or Fab from either Ipilimumab or L3D10 (10 μg/ml) for 4 h. **e** Dose–response curve depicting inhibition of B7-1 trans-endocytosis by L3D10 and Ipilimumab Fab. As in **d**, except varying doses of control hIgG-Fc or Fab were added to the co-culturing. **f** As in **d**, except that B7-2-GFP-transfected CHO cells were used. **g** Dose–response curve depicting inhibition of B7-2 trans-endocytosis by L3D10 and Ipilimumab Fab. As in **e**, except that B7-2-GFP-transfected CHO cells were used. Data shown (mean ± S.D.) are % of trans-endocytosis over varying doses of Fab. All assays have been repeated at least three times

It has been demonstrated that CTLA-4 mediates trans-endocytosis of cell surface B7-2.^12^ These findings provide us with another assay to measure the blocking activity of anti-CTLA-4 mAbs under more physiologically relevant conditions. We used CHO cells transfected with either GFP-tagged B7 or OFP-tagged CTLA-4 (Fig. 4a). The use of fluorescent-protein-tagged receptor and ligand allowed us to quantify their interaction in live cells. To ensure CTLA-4-OFP^+^ cells are surrounded by B7-2-GFP^+^ cells, we added excess amounts of B7-2-GFP^+^ cells. As shown in Fig. 4b, co-incubation at 37 °C resulted in a time-dependent accumulation of a new population of cells that expressed both CTLA-4 and B7-2. This accumulation peaked at 4 h after co-incubation. Since with time, essentially all OFP^+^ cells had become OFP^+^GFP^+^ whereas the percentage of GFP^+^OFP^−^ cells remained unchanged throughout the co-incubation, the appearance of double-positive cells was due to uptake of B7-2-GFP by CTLA-4-OFP-transfected CHO cells, as expected. Consistent with this interpretation, the scatters of the OFP^+^GFP^+^ are those of single cells (Supplementary information, Figure S3C). As control, we co-cultured the CTLA-4-OFP transfectants with the B7-H2-GFP transfectants. As shown in Fig. 4c, no detectable transfer of GFP signal to the CTLA-4-OFP transfectant was observed over a 4 h period, thus confirming the specificity of the assay. Having established the model, we compared the effect of L3D10 and Ipilimunab Fabs for their impact on trans-endocytosis. As shown in Fig. 4d, e, the L3D10 Fab is ~10-fold more efficient than the Ipilimumab Fab in blocking trans-endocytosis of B7-1. Similarly, the L3D10 Fab is ~30-fold more effective in blocking B7-2 trans-endocytosis (Fig. 4f, g). It should be noted that, whereas L3D10 Fab effectively blocked trans-endocytosis of B7-2 (IC_50_ = 1 μg/ml), Ipilimumab Fab achieved <20% inhibition of B7-1 trans-endocytosis (Fig. 4e) and only 30% inhibition of B7-2 trans-endocytosis (Fig. 4g) when used at 10 μg/ml. By molar ratio, this dose is equal to 30 μg/ml of intact Ipilimumab, which is ~50% higher than the steady-state plasma drug concentration when an effective dose of Ipilimumab (3 mg/kg) is used in clinic.^7^

### Ipilimumab does not block downregulation of B7-1/B7-2 by CTLA-4 in vivo

**Fig. 5:**
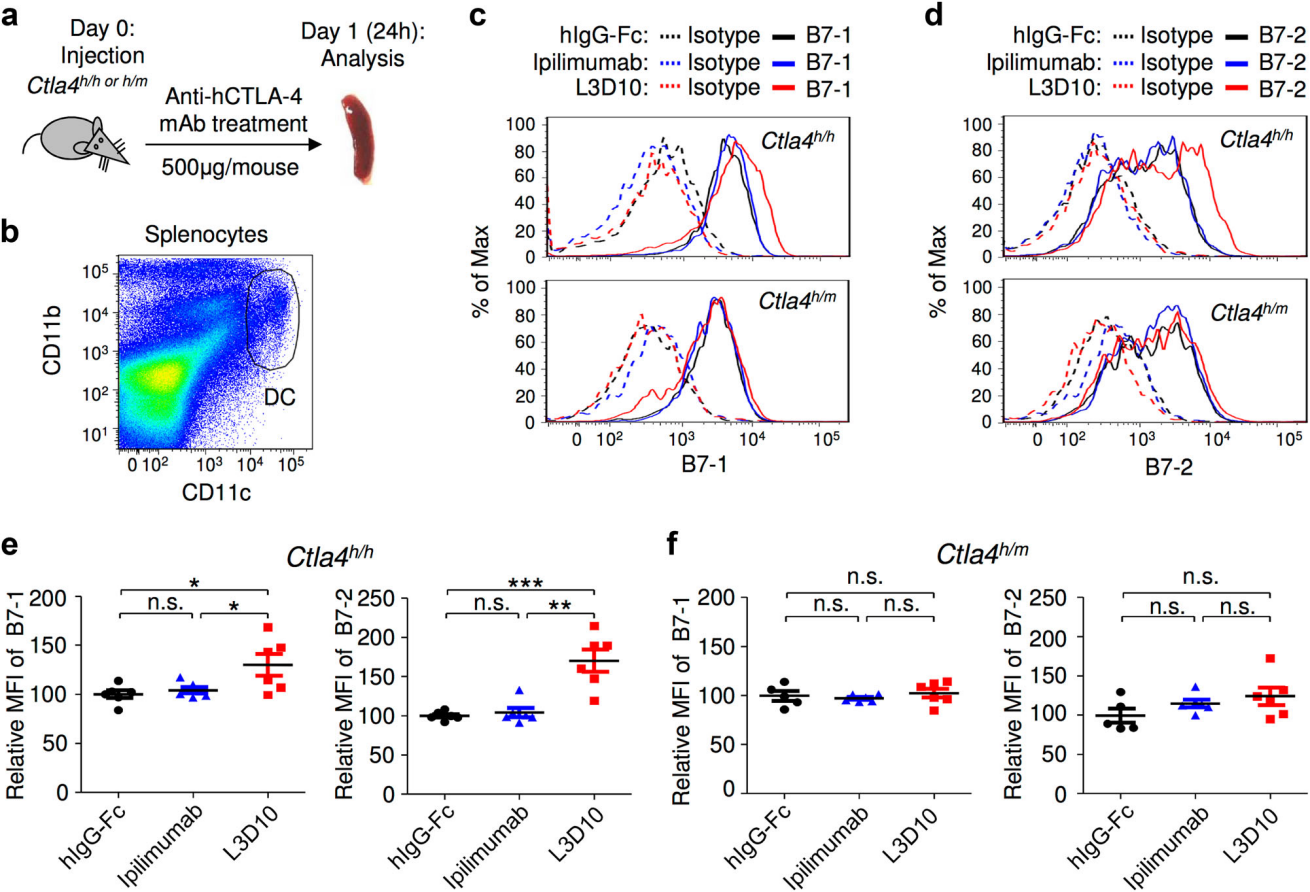
Ipilimumab does not block B7-CTLA-4 interaction in vivo. **a** Diagram of experimental design. **b** Representative data showing the phenotype of CD11b^+^CD11c^high^ DC analyzed for B7 expression. **c** Representative histograms depicting the levels of mB7-1 on DC from mice that received control hIgG-Fc, L3D10 or Ipilimumab. Data in the top panel show antibody effect in homozygous human *CTLA4* knock-in mice (*Ctla4*^*h/h*^), while that in the bottom panel show antibody effect in the heterozygous mice (*Ctla4*^*h/m*^). **d** As in **c**, except that expression of mB7-2 is shown. Data shown in **c** and **d** are representative of those from three mice per group and have been repeated once. **e** In human *CTLA4* homozygous mice, L3D10 but not Ipilimumab-induced upregulation of mB7-1 (left panel) and mB7-2 (right panel). Data shown (mean ± S.E.M.) are summarized from two experiments involving a total of six mice per group. **f** As in **e**, except that heterozygous mice are used. Neither L3D10 nor Ipilimumab blocks B7-CTLA-4 interaction in mice that co-dominantly express both mouse and human *Ctla4* genes. Statistical significance was determined using Student’s *t* test. *P < 0.05, **P < 0.01, ***P < 0.001. n.s., not significant

CTLA-4 is expressed predominantly in Treg cells where it suppresses autoimmune diseases by downregulating B7-1 and B7-2 expression on DCs^26^ among other potential mechanisms. Since targeted mutation of *Ctla4*^26^ and treatment with blocking anti-CTLA-4 mAb^12^ both increase expression of B7-1 and B7-2 on DCs, it has been suggested that the physiological function of CTLA-4 on Treg cells is to downregulate B7 on DCs through trans-endocytosis.^12,27^ Therefore, a direct consequence of blocking the B7-CTLA-4 interaction is the upregulation of B7 in DCs. To evaluate blocking activities of anti-CTLA-4 mAbs in vivo, we injected very high doses of anti-CTLA-4 mAb (500 μg/mouse, which is roughly 25 mg/kg or greater than eight times the highest Ipilimumab dose used in clinics, 3 mg/kg) into *Ctla4*^*h/h*^ or *Ctla4*^*h/m*^ mice and collected spleen cells to measure levels of B7-1 and B7-2 on CD11c^high^ DCs 24 h after injection (Fig. 5a, b). In comparison to *Ctla4*^*h/h*^ mice that received human IgG1 Fc, DCs from L3D10-treated mice showed a modest but statistically significant elevation of B7-1 and a robust upregulation of B7-2 (Fig. 5c–e). The magnitude of upregulation in B7-2 is comparable to what was achieved using a blocking anti-CTLA-4 mAb in co-cultured human Treg cells and DCs.^12,27^ On the other hand, Ipilimumab failed to upregulate B7-1 and B7-2 in vivo. To rule out the potential effect of contaminating LPS, we measured the endotoxin levels in our antibody preparations and found that they were between 0.00025 and 0.0025  g/μg, a level 2–10-fold lower than in the IgG-Fc controls, which did not cause B7-1 and B7-2 upregulation in vivo (Supplementary information, Figure S4).

Since at least 50% of the CTLA-4 proteins in the *Ctla4*^*h/m*^ mice are of mouse origin and do not bind to the anti-human CTLA-4 antibodies (Supplementary information, Figure S5) but functionally cross-react with mouse B7-1 and B7-2,^28–30^ and because trans-endocytosis should only require some unblocked CTLA-4 molecules on Treg cells, the unbound CTLA-4 should downregulate B7 on DCs even in the presence of blocking anti-human CTLA-4 mAbs. Indeed, neither antibody caused upregulation of B7-1 and B7-2 on DCs from *Ctla4*^*h/m*^ mice (Fig. 5c, d, f). The upregulation of B7 by L3D10 specifically in the *Ctla4*^*h/h*^ but not in the *Ctla4*^*h/m*^ mice further validates the notion that B7 upregulation depends on complete blocking of the B7-CTLA-4 interaction and rules out the possibility that upregulation of B7 by L3D10 is due to contaminating LPS.

**Fig. 6:**
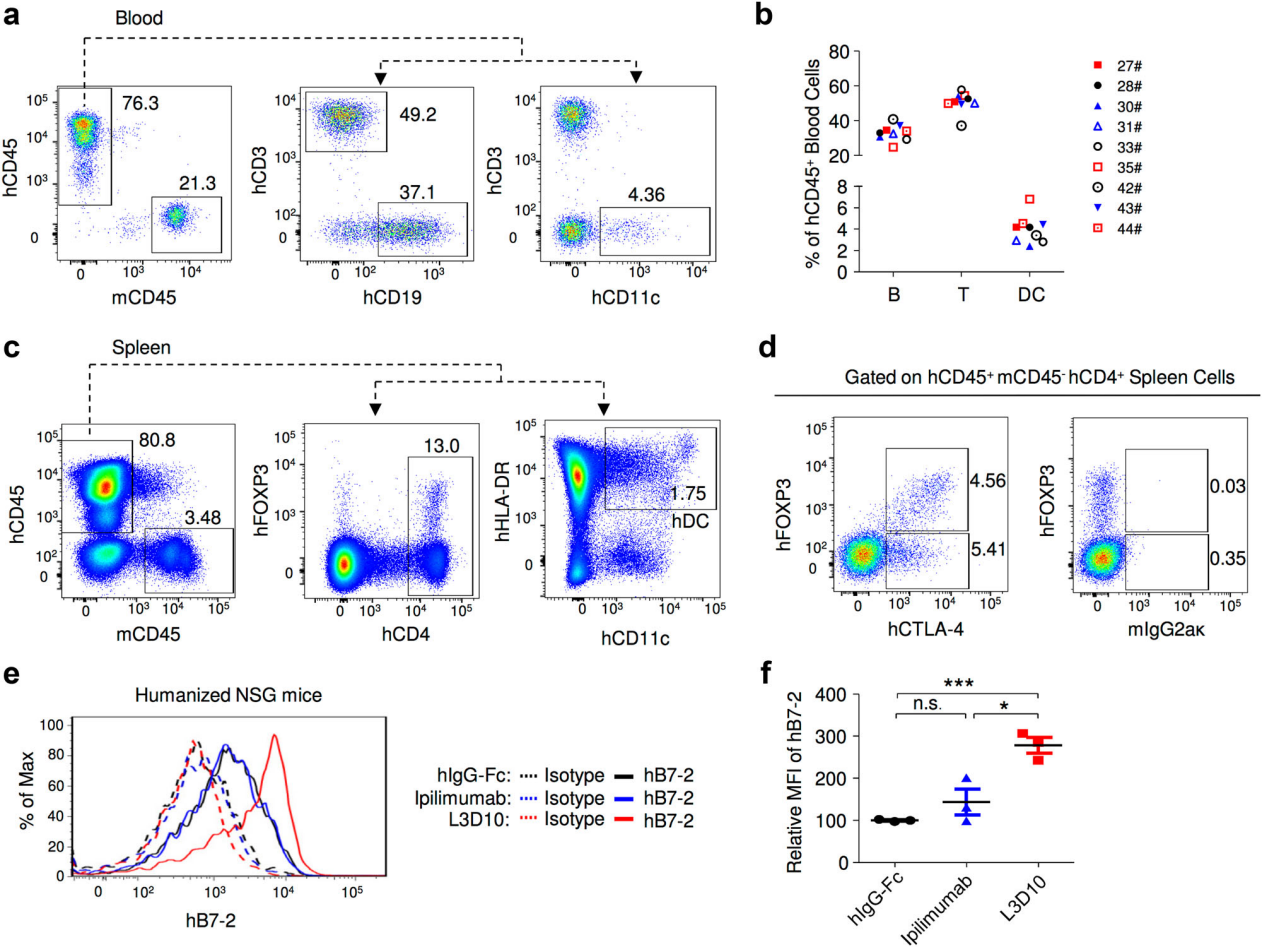
Ipilimumab does not block human B7-human CTLA-4 interaction in vivo. **a** FACS profiles depicting composition of human leukocytes among the peripheral blood leukocytes (PBL) of NSG^TM^ mice reconstituted with human cord blood CD34^+^ cells. **b** Summary data of individual mice as analyzed in **a**. **c** Normal composition of Treg cells and DCs in spleen of humanized NSG^TM^ mice. **d** Expression of FOXP3 and CTLA-4 among human CD4 T cells in mice spleen. **e**, **f** L3D10 but not Ipilimumab blocks human B7-2-human CTLA-4 interaction in the human cord blood CD34^+^ stem cell-reconstituted NSG™ mice. The humanized mice received intraperioneal treatment of either control Ig or anti-CTLA-4 mAbs (500 μg/mouse). Splenocytes were harvested at 24 h after injection and analyzed for expression of B7 on DC. **e** Representative profiles of hB7-2 on DC. **f** Summary data (mean ± S.E.M.) from two independent experiments. The mean data in the control mice are artificially defined as 100 and those in experimental groups are normalized against the control. Statistical significance was determined using Student’s *t* test. *P < 0.05, **P < 0.01, ***P < 0.001. n.s., not significant

To determine if the lack of blocking by Ipilimumab observed in the *Ctla4*^*h/h*^ mice can be observed between human T cells and human DCs, we employed human cord blood CD34^+^ stem cell-reconstituted NSG™ mice. As shown in Fig. 6a, b, the peripheral blood of the mice consisted of 70–90% of human leukocytes, including T and B lymphocytes and DCs. In the spleen, high frequencies of FOXP3^+^ Treg cells and CD11c^+^HLA-DR^+^ DCs were observed (Fig. 6c). Significant expression of hB7-2 (Fig. 6e) but not hB7-1 (data not shown) was observed on DCs. Since human CTLA-4 was expressed at high levels in FOXP3^+^ Treg cells (Fig. 6d), we used this model to study the B7-2-CTLA-4-interaction between human DCs and Treg cells in an in vivo setting. We found that Ipilimumab did not significantly upregulate B7-2 expression on DCs (*P* = 0.22), whereas DCs from L3D10-treated mice showed nearly 2.5-fold higher levels of B7-2 (*P* < 0.001) (Fig. 6e, f), consistent with human *CTLA4* gene knock-in mouse data. Therefore, L3D10 but not Ipilimumab blocks the downregulation of B7-2 by hCTLA-4 in the human hematopoietic system of humanized mice.

### Blocking the B7-CTLA-4 interaction is required for neither Treg depletion nor tumor rejection

**Fig. 7:**
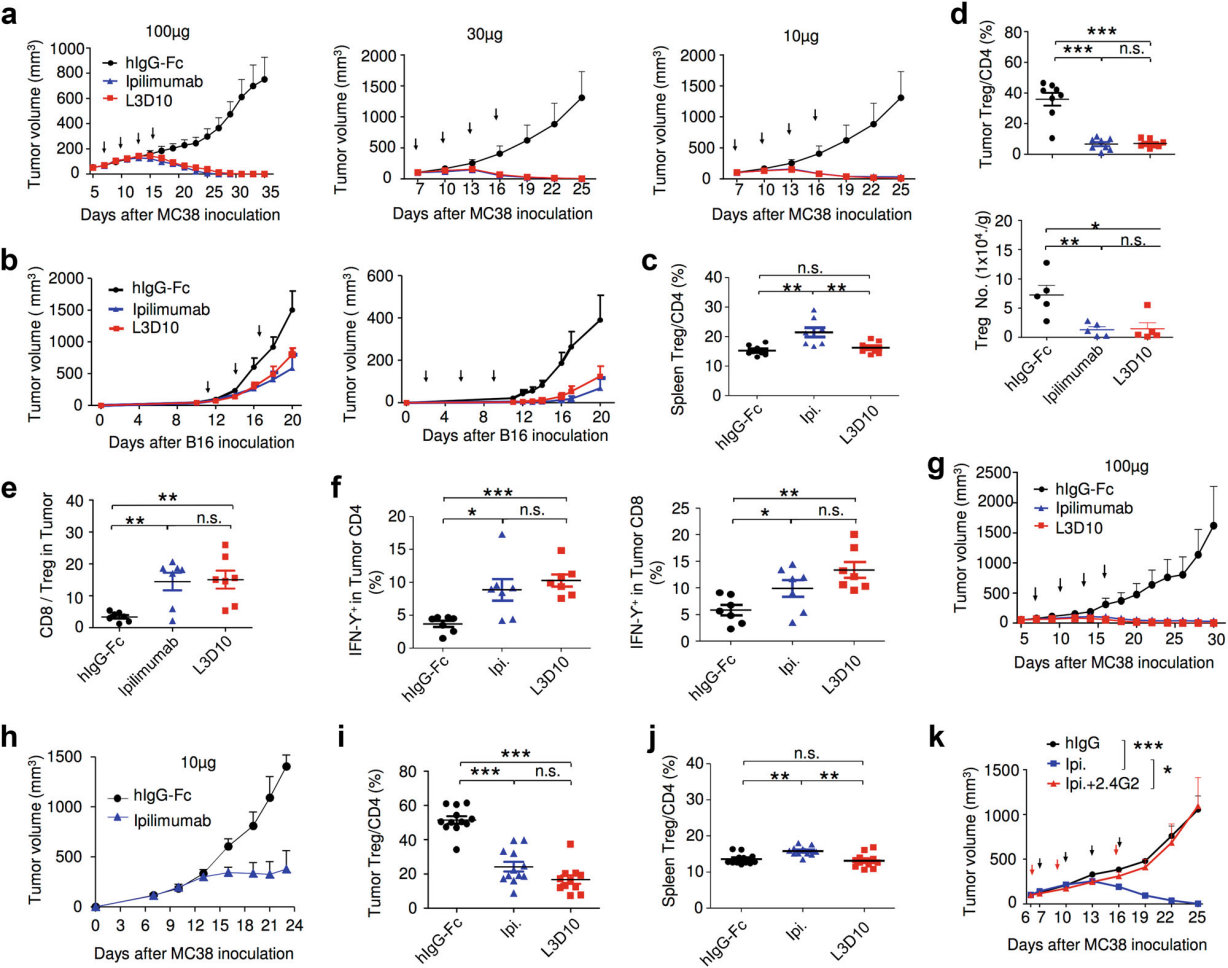
Blocking the B7-CTLA-4 interaction does not contribute to anti-CTLA-4 mAbs elicited cancer immunotherapeutic activity and intratumorial Treg depletion. **a** Comparable immunotherapeutic effect despite vastly different blocking activity by two anti-CTLA-4 mAbs. 5 × 10^5^ or 1 × 10^6^ MC38 tumor cells were injected (s.c.) into *Ctla4*^*h/h*^ mice (*n* = 5–6), and mice were treated (i.p.) with 100 μg (left), 30 μg (middle) or 10 μg (right) Ipilimumab, L3D10 or control hIgG-Fc per mouse on days 7, 10, 13 and 16, as indicated by arrows. Data represent mean ± S.E.M. of 5–6 mice per group. Statistical analyses were performed by two-way repeated measures ANOVA (treatment × time). For 100 μg treatments, Ipilimumab vs hIgG-Fc: *P* < 0.0001; L3D10 vs hIgG-Fc: *P* < 0.0001; Ipilimumab vs L3D10: *P* = 0.0699. For 30 μg treatments, Ipilimumab vs hIgG-Fc: *P* < 0.0001; L3D10 vs hIgGFc: *P* < 0.0001; Ipilimumab vs L3D10: *P* = 0.9969. For 10 μg treatments, Ipilimumab vs hIgG-Fc: *P* < 0.0001; L3D10 vs hIgG-Fc: *P* < 0.0001; Ipilimumab vs L3D10: *P* = 0.9988. Data are representative of 3–5 independent experiments. **b** Ipilimumab and L3D10 have similar therapeutic effect for B16 melanoma growth. 1 × 10^5^ B16 tumor cells were injected (s.c.) into *Ctla4*^*h/h*^ mice (*n* = 4–5), and mice were treated (i.p.) with 100 μg (left) or 250 μg (right) Ipilimumab, L3D10 or control hIgG-Fc on day 11, 14, 17(left) or on day 2, 5 and 8 (right), as indicated by arrows. For the left panel, Ipilimumab vs hIgG-Fc: *P* = 0.0265; L3D10 vs hIgG-Fc: *P* = 0.0487; Ipilimumab vs L3D10: *P* = 0.302. For the right panel, Ipilimumab vs hIgG-Fc: *P* = 0.00616; L3D10 vs hIgG-Fc: *P* = 0.0269: Ipilimumab vs L3D10: *P* = 0.370, Data represent mean ± S.E.M. of 4–5 mice per group. **c**–**f** Blocking B7-CTLA-4 interaction does not contribute to selective depletion of Treg cells in tumor microenvironment in the *Ctla4*^*h/h*^ mice. L3D10 and Ipilimumab did not delete Treg cells in the spleen (**c**) of mice at 3 days after third treatment. Data shown are the percentage of Foxp3^+^ cells among CD4 T cells in *Ctla4*^*h/h*^ mice. *n* = 6 mice for each group. Both L3D10 and Ipilimumab depleted Treg cells in tumors transplanted into the *Ctla4*^*h/h*^ mice, as determined by % Treg cells among CD4 T cells (**d**, upper), absolute Treg cell number (**d**, lower) and CD8/Treg ratios (**e**). Summary data from two experiments involving seven mice per group are presented in **d** (upper panel) and **e**. The numbers of Foxp3^+^ cells (**d**, lower panel) in the tumor from *Ctla4*^*h/h*^ mice were counted by flow cytometry on 3 days after the third antibody treatment. *n* = 5 for each group. Statistical analyses were performed by ordinary one-way ANOVA with Tukey’s multiple comparisons test. **f** Blocking B7-CTLA-4 interaction does not contribute to increased IFNγ-producing cells among tumor-infiltrating CD4 (left) or CD8 (right) T cells. Summary data are from two experiments involving seven mice per group. Single-cell suspensions of collagenase-digested tumors were prepared between 13 or 16 days and cultured in the presence of Golgi blocker for 4 h and stained for intracellular cytokines. **g**–**j** In *Ctla4*^*h/m*^ mice where neither antibody blocks the B7-CTLA-4 interaction, both L3D10 and Ipilimumab induce robust tumor rejection and intratumorial Treg depletion. As in **a**, except that heterozygous mice that express both mouse and human CTLA-4 were used. **g**, **h** Both higher doses (**g**, 100 μg/mouse/ injection) and lower doses (**h**, 10 μg/mouse/injection) of antibody treatments showed effective therapeutically effects. In **g**, Ipilimumab vs hIgG-Fc: *P* < 0.0001; L3D10 vs hIgG-Fc: *P* < 0.0001; Ipilimumab vs L3D10: *P* = 0.4970. Data are representative of five independent experiments. Treg cells were selectively depleted in the tumor (**i**) but not in the spleen (**j**) of *Ctla4*^*h/m*^ mice that neither antibodies significantly blocked B7-CTLA-4 interaction in vivo. Data (mean ± S.E.M.) shown in **c**, **d**, **e** and **i** are the percentage of Treg cells at 18 (experiment 1) or 20 days (experiment 2) after tumor cell challenge and 11 or 13 days after initiation of 3 or 4 anti-CTLA-4 mAb treatments as indicated in arrows. Statistical significance in **c**–**f** and **i**, **j** was determined using Mann–Whitney test. **k** Anti-FcR mAb administration abrogated the therapeutic effect of Ipilimumab. 5 × 10^5^ MC38 tumor cells were injected (s.c.) into *Ctla4*^*h/h*^ mice, and mice were treated (i.p.) with 30 μg Ipilimumab alone, or 30 μg Ipilimumab (black arrow) plus 1 mg 2.4G2 (red arrow) or control hIgG-Fc on days 7, 10, 13 and 16, as indicated. Statistical analyses were performed by two-way repeated measures ANOVA (treatment × time). Ipilimumab vs hIgG-Fc: *P* = 0.0003; Ipilimumab plus 2.4G2 vs hIgG-Fc: *P* = 0.6962; Ipilimumab plus 2.4G2 vs Ipilimumab: *P* = 0.0259

To test whether blockade of the B7-CTLA-4 interaction is required for immunotherapeutic effect, we first compared L3D10 and Ipilimumab for their ability to induce tumor rejection. We challenged the *Ctla4*^*h/h*^ mice with the colon cancer cell line MC38. When tumors reached a size of ~5 mm in diameter, the mice were treated four times with control human IgG-Fc, L3D10 or Ipilimumab at doses of 10, 30 and 100 μg/mouse/injection and tumor size was observed for 4–6 weeks. Whereas tumors grew progressively in the control IgG-Fc-treated mice, complete rejection was achieved by both anti-CTLA-4 mAbs, even when as little as 10 μg/mouse was used (Fig. 7a). In multiple experiments, treatment with either of the two antibodies gave comparable tumor rejection. In another tumor model, B16 melanoma, both antibodies induced similar retardation of tumor growth, regardless of whether the antibodies were administered prior to or after the tumor was established (Fig. 7b). However, complete rejection was not achieved by antibody monotherapy, as expected from previously published studies.^10^

Recent studies have demonstrated that the therapeutic efficacy of anti-mouse Ctla-4 mAbs is affected by the Fc subclass and host Fc receptor, which in turn affect antibody-dependent depletion of Treg cells selectively within the tumor microenvironment.^9–11^ However, it has not been tested whether such depletion requires blockade of the B7-CTLA-4 interactions. This remains possible as such a blockade can upregulate B7 (Figs. 5 and 6), which could cause supra-stimulation of CD28, potentially causing T cell apoptosis.^31,32^ To address this issue, we killed tumor-bearing mice before the rejections were complete and analyzed the frequency of Treg cells in mice that received control Ig, Ipilimumab or L3D10. Whereas neither anti-CTLA-4 antibody reduced Treg cell numbers in the spleen (Fig. 7c), both did in the tumor microenvironment, based on the percentile (Fig. 7d, upper panel) and absolute numbers (Fig. 7d, lower panel) of Treg cells. Interestingly, although tumor-infiltrating Foxp3^−^ T cells expressed CTLA-4, albeit at lower levels, they were not depleted by anti-CTLA-4 mAbs (Supplementary information, Figure S6A). The efficient depletion of Treg cells in tumors but not in spleen or lymph node can be explained by the much higher expression of CTLA-4 on tumor infiltrated Treg cells (Supplementary information, Figure S6B and C), which is also reported by previous studies.^9–11^ As a result of Treg cell depletion, the ratio of CD8 T cells over Treg cells was selectively increased in the tumor (Fig. 7e). Moreover, depletion of Treg cells was associated with functional maturation of CD8 and CD4 T cells, as demonstrated by increased interferon γ-producing cells (Fig. 7f) and tumor necrosis factor α-producing T cells within tumor microenvironment (Supplementary information, Figure S7A and B) but not in the spleen (Supplementary information, Figure S7C-F).

Since L3D10 and Ipilimumab are comparable in the ability to deplete Treg cells in the tumor microenvironment, blockade of the B7-CTLA-4 interaction unlikely contributes to Treg depletion. In addition, since Ipilimumab does not appear to block the B7-CTLA-4 interaction in vivo and still confers therapeutic effect in the *Ctla4*^*h/h*^ mice and in melanoma patients, blockade of this interaction is unlikely required for its therapeutic effect. Furthermore, since two mAbs with drastically different blocking activities have comparable therapeutic effects and show similar efficacy in selective Treg depletion in tumor microenvironment, blocking the B7-CTLA-4 interaction does not enhance the therapeutic effect of an antibody. To substantiate this observation, we tested the therapeutic response of the two anti-CTLA-4 mAbs in the *Ctla4*^*h/m*^ mice in which the anti-human CTLA-4 mAbs can bind to a maximum of 50% of CTLA-4 molecules and in which neither antibody can block the B7-CTLA-4 interaction to achieve upregulation of B7 on DCs (Fig. 5f). Again, both antibodies caused rapid rejection of the MC38 tumors when high doses (Fig. 7g) or lower doses (Fig. 7h) of antibodies were used. Correspondingly, both antibodies selectively depleted Treg cells in tumor microenvironment (Fig. 7i) but not in the spleen (Fig. 7j). These genetic data further question the relevance of CTLA-4 blockade in both tumor rejection and local Treg depletion and dispute the prevailing hypothesis that anti-CTLA-4 mAb induces cancer immunity through blocking the B7-CTLA-4 interaction.^4^ Since the therapeutic antibodies were all efficient in Treg depletion but varied in their ability to block the B7-CTLA-4 interaction, we hypothesized that these antibodies caused tumor rejection by inducing Treg depletion through antibody-dependent cellular cytotoxicity (ADCC). Since ADCC is dependent on FcR on the host effector cells, we tested if anti-FcR antibodies can abrogate tumor rejection. As shown in Fig. 7k, concurrent anti-FcR treatment completely erased the tumor immunotherapeutic effect of Ipilimumab.

**Fig. 8:**
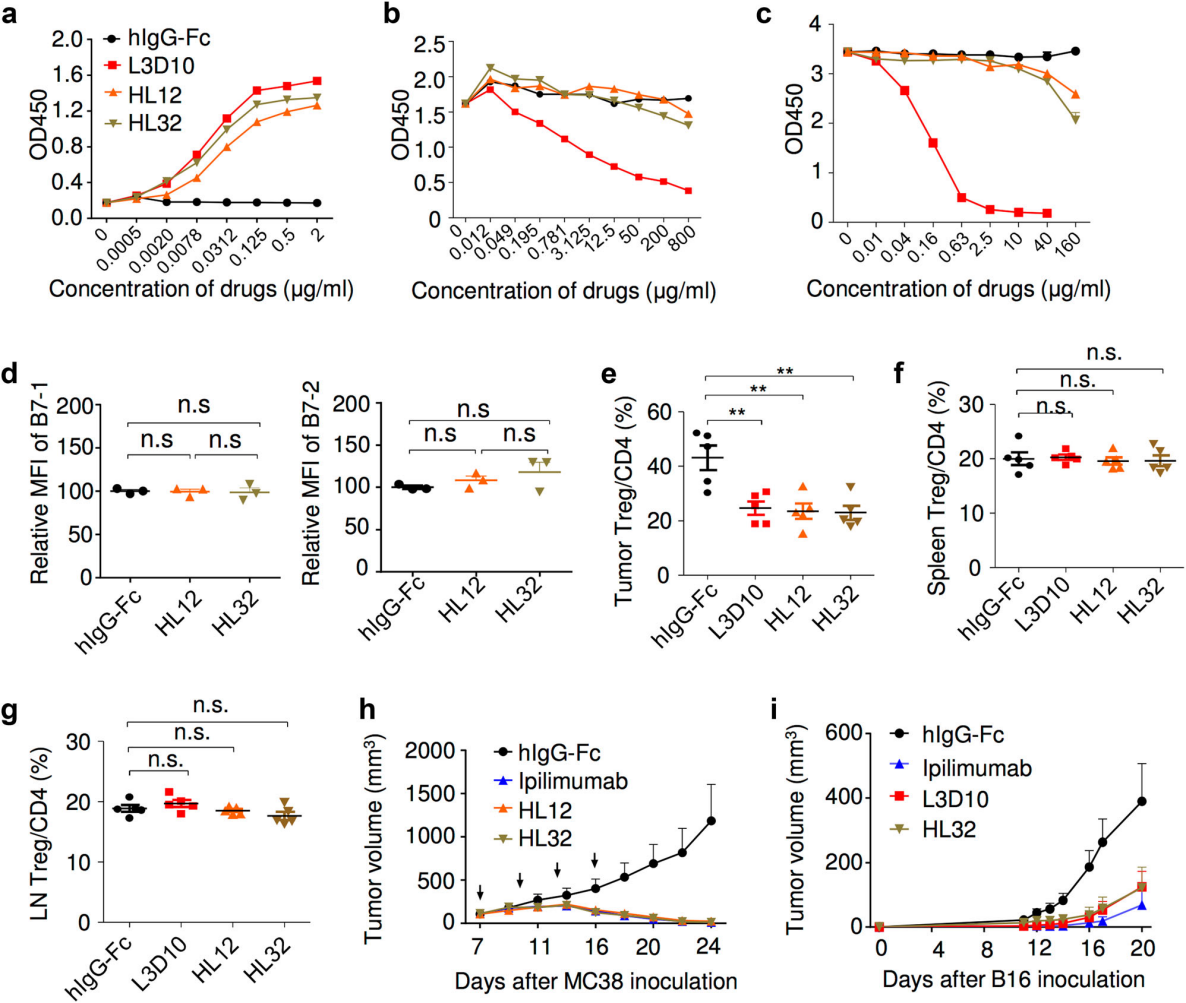
Humanized L3D10 progenies (HL12 and HL32) that lost blocking activities remain effective in local Treg depletion and tumor rejection. **a** Binding activities of HL12, HL32 and L3D10 to 1 μg/ml immobilized polyhistidine-tagged CTLA-4. **b** HL12 and HL32 failed to block the B7-1-CTLA-4 interaction. B7-1-Fc was immobilized at a concentration of 0.5 μg/ml. Biotinylated CTLA-4-Fc was added at 0.25 μg/ml along with grading concentration of anti-CTLA-4 mAbs. **c** HL12 and HL32 barely block the B7-2-CTLA-4 interaction. As in **b**, except B7-2-Fc is immobilized. **d** HL12 and HL32 failed to upregulates B7-1 and B7-2 in vivo. As in Fig. 5, *Ctla4*^*h/h*^ mice received 500 μg/ mouse/injection of control hIgG-Fc or anti-CTLA-4 mAbs. Spleen cells were collected next day to determine levels of B7-1 and B7-2 on CD11b^+^CD11c^high^ DCs, as detailed in Fig. 5. *n* = 3 for each group. **e**–**g** Similar as L3D10, HL12 and HL32 showed selective depletion of Treg cells in the tumor microenvironment in the *Ctla4*^*h/h*^ mice. As in Fig. 7, L3D10, HL12 and HL32 elicited comparable and efficient depletion of Treg cells in tumor (**e**), but did not deplete Treg cells in spleen (**f**) and tumor-draining lymph node (**g**). Data shown were pooled from two experiments. *n* = 5 mice for each group. Mice were killed 1 day after one injection of 100 μg indicated drug. **h** Efficient rejection of MC38 tumors by Ipilimumab and humanized L3D10 antibodies HL12 and HL32. Mice-bearing MC38 were treated on days 7, 10, 13 and 16 days after tumor cells inoculation with 100 μg control IgG-Fc or Ipilimumab or HL12, HL32. Data shown are means and S.E.M. of tumor volume. *n* = 6 mice for each group. Statistical analyses were performed by two-way repeated measures ANOVA (treatment × time). Ipilimumab vs hIgG-Fc: *P* = 0.034; HL12 vs hIgG-Fc: *P* = 0.037; HL32 vs hIgG-Fc: *P* = 0.0336; HL12 vs Ipilimumab : *P* = 0.9021; HL32 vs Ipilimumab : *P* = 0.9972 ; HL32 vs HL12: *P* = 0.7250. **i** HL32 and L3D10 are comparably effective in treatment of B16 tumor cells in a minimal disease model. 1 × 10^5^ B16 tumor cells were injected (s.c.) into *Ctla4*^*h/h*^ mice (*n* = 4–5), and mice were treated (i.p.) with 250 μg of Ipilimumab, L3D10, HL32 or control IgG-Fc on days 2, 5 and 8, as indicated by arrows. HL32 vs hIgG-Fc: *P* = 0.0002; L3D10 vs HL32: *P* = 0.9998; Ipilimumab vs HL32: *P* = 0.8899. Data represent mean ± S.E.M. of 5–6 mice per group

During humanization of the L3D10 mAb, we obtained two clones called HL12 and HL32 that retained potent binding to CTLA-4 (Fig. 8a) but lost the ability to block CTLA-4 binding to plate-bound B7-1 (Fig. 8b) and B7-2 (Fig. 8c), perhaps due to an approximately fourfold increase in off-rate and correspondingly bivalent avidity (Supplementary information, Table S1). These antibodies also lost the ability to induce upregulation of B7-1 and B7-2 on host APC (Fig. 8d). The ability of the antibodies to block soluble B7 binding to immobilized CTLA-4-Fc was also abrogated (Supplementary information, Figure S8A). The fact that these humanized antibodies have lost the ability to block the B7-CTLA-4 interaction provides us with an opportunity to further test whether the blocking activity is essential for tumor rejection and Treg depletion. As shown in Fig. 8e, despite the loss of blocking activity, the humanized antibodies rapidly induced Treg depletion in the tumor microenvironment but not in the spleen (Fig. 8f) or draining lymph nodes (Fig. 8g). Furthermore, HL12 and HL32 exhibited similar effects as L3D10 on the abundance of T cell subpopulations in peripheral lymph organs and tumors (Supplementary information, Figure S8B and C). More importantly, both antibodies were as effective as Ipilimumab and parental L3D10 in causing rejection of MC38 (Fig. 8h) and B16 (Fig. 8i) tumors.

### B7-CTLA-4 interaction is not required for the immunotherapeutic activity of Ipilimumab

**Fig. 9:**
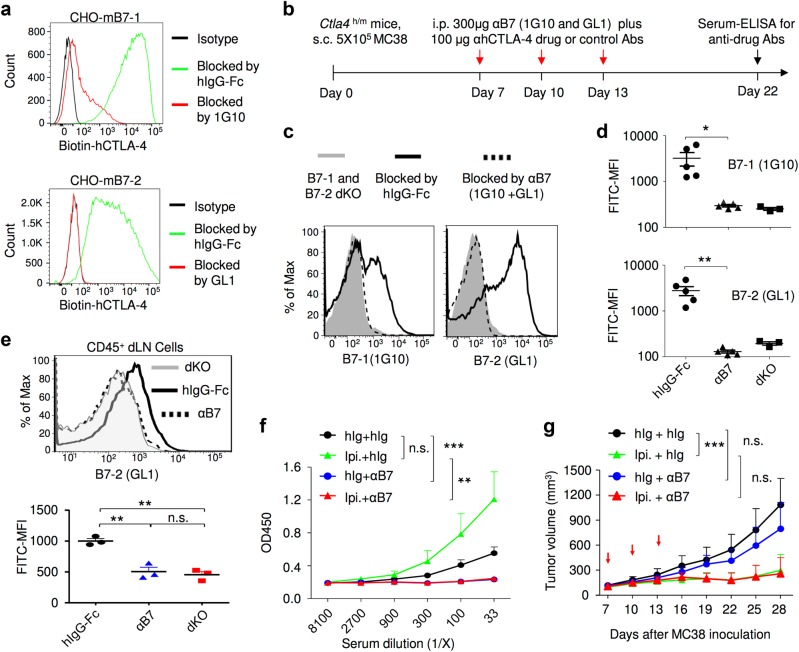
Therapeutic effect of Ipilumumab is not achieved by blocking CTLA-4-B7 negative signaling. **a** Confirmation of the blocking activities of anti-B7 mAbs. CHO cells expressing mouse B7-1 or B7-2 were incubated with a mixture of antibodies (20 μg/ml) and biotinylated human CTLA-4-Fc (2 μg/ml) for 1 h. After washing away unbound proteins, the cell surface CTLA-4-Fc was detected by PE-conjugated streptavidin and measured by flow cytometry. Data shown are representative FACS profiles and have been repeated two times. **b** Diagram of experimental design. MC38 tumor-bearing *Ctla4*^*h/m*^ mice received anti-B7-1 and anti-B7-2 antibodies (300 μg/mouse/injection, once every 3 days for a total of three injections) in conjunction with either control Ig or Ipilimumab, mice that received Ipilimumab without anti-B7-1 and anti-B7-2 were used as positive control for tumor rejection. **c**, **d** Saturation of B7-1 and B7-2 by antibody treatments as diagrammed in **b**. The PBL were stained with FITC-conjugated anti-B7-1 and anti-B7-2 mAbs at 24 h after the last anti-B7 treatment on day 13. PBL from *Cd80*^−/−^*Cd86*^−/−^ mice were used as negative control. **e** Complete blocking of B7-2 in vivo. As in **c** and **d**, except that CD45^+^ leukocytes were gated from single-cell suspensions of draining lymph nodes in mice-bearing MC38 were used. The top panel depicts profiles of B7-2 staining, while the lower panel shows the mean fluorescence intensities. This study has been repeated three times. **f** Ablation of antibody responses confirmed the functional blockade of B7 by anti-B7-1 and anti-B7-2 mAbs. Sera were collected at day 22 after tumor challenge to evaluate anti-human IgG antibody response. **g** Saturating blocking by anti-B7-1 and anti-B7-2 mAbs does not affect immunotherapeutic effect of Ipilimumab. Data shown in **g** are tumor volumes over time and have been repeated twice with similar results. Data in **d**–**g** represent mean ± S.E.M.; n.s., not significant

A critical prediction of the CTLA-4 checkpoint blockade hypothesis is that anti-CTLA-4 mAb should not confer an immunotherapeutic effect unless B7 is present to deliver a negative signal. Since mice with targeted mutations of *Cd80* (encoding B7-1) and *Cd86* (encoding B7-2) do not have Treg cells^33^ and thus express very little Ctla4, we tested this prediction by using a saturating dose of anti-B7-1 (1G10) and anti-B7-2 (GL1) mAbs, which block the binding of human CTLA-4 to mB7-1 and mB7-2, respectively (Fig. 9a). As diagrammed in Fig. 9b, we treated MC38 tumor-bearing mice with Ipilimumab in conjunction with either control Ig or a combination of anti-mB7-1 and anti-mB7-2 mAbs. The anti-mB7 mAbs used completely masked all B7-1 and B7-2 in the peripheral blood leukocytes as their binding to new anti-mB7 mAb was reduced to that observed in mice having targeted mutations of both *Cd80* and *Cd86* (dKO) (Fig. 9c, d). Similar blocking of B7-2 was observed for DCs from tumor-draining lymph nodes (Fig. 9e). However, the levels of B7-1 were barely detectable by 1G10 mAb regardless of antibody treatment (data not shown), making it impossible to evaluate the extent of masking of endogenous B7-1. Since B7-1 and B7-2 are both required for the antibody response to antigens,^34^ and since anti-CTLA-4 antibodies are potent inducers of anti-drug antibodies (ADA),^5^ ADA is a good indicator for function of both B7-1 and B7-2 in vivo. Functional blocking was further confirmed by the fact that the antibody response to Ipilimumab was completely abrogated (Fig. 9f). Importantly, a saturating blockade of B7 did not affect the Ipilimumab-induced tumor rejection as anti-mB7 and control Ig-treated mice were equally responsive to Ipilimumab therapy (Fig. 9g). Therefore, abrogation of negative signaling by B7 does not explain immunotherapeutic effect of Ipilimumab.

**Fig. 10:**
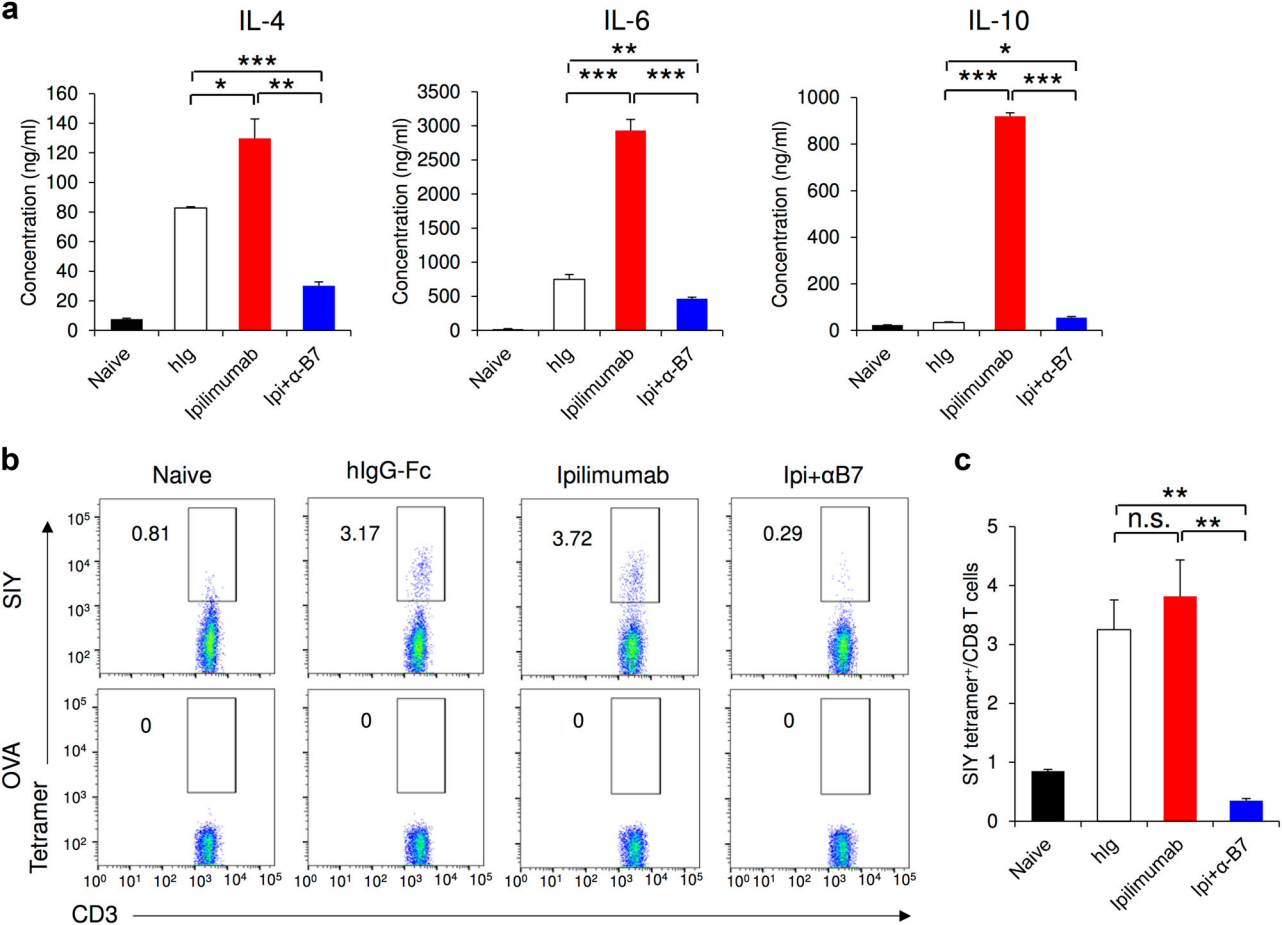
In vivo treatment of anti-B7 mAbs prevents Ipilimumab-mediated T cell activation and de novo priming of CD8 T cell. **(a)** Functional blockade of B7 by anti-B7-1 (1G10) and anti-B7-2 (GL1) mAbs prevented Ipilimumab-induced CD4 T cell activation. As in Fig. 9, MC38 tumor-bearing *Ctla4*^*h/h*^ mice (*n* = 5 for each group) were treated intraperitoneally with hIgG-Fc (100 μg/mouse/injection), Ipilimumab (100 μg/mouse/injection) or Ipilimumab plus anti-mB7 mAbs (300 μg 1G10 plus 300 μg GL1/mouse/injection) on day 7, 10 and 13 and killed on day 14. Sex and age-matched, tumor-free *Ctla4*^*h/h*^ mice were used as control naive mice. Spleen T cells from these mice were purified by MACS negative selection and co-cultured with naive spleen DCs in the presence of 10 μg/ml hIgG-Fc for 4 days. The levels of Th2 cytokines (including IL-4, IL-6 and IL-10) in the supernatant were quantitated by cytokine beads assays (CBA). **b**, **c** Anti-B7 mAbs prevented Ipilimumab-induced priming of antigen-specific CD8 T cells. As in **a**, except that all mice (*n* = 4 for each group) were immunized subcutaneously with 50 μg SIY peptide emulsified in 100 μl Complete Freund’s Adjuvant (CFA) on day 8. Mice were killed on day 15 and tumor-draining lymph nodes were collected to evaluate SIY-specific CD8 T cells (gated on CD3^+^ CD4^−^ cells) by tetramer staining. OVA tetramer was used for control staining. Representative FACS profiles (**b**) and summary data (**c**) are shown. Data shown are representative of two independent experiments with similar results

Another key prediction of the checkpoint blockade hypothesis is that anti-CTLA-4 mAb releases breaks of naive T cells to achieve cancer immunotherapeutic effect. Since anti-B7 mAbs completely abrogated T cell-dependent antibody responses, we tested if the in vivo treatment of anti-B7 mAbs prevented Ipilimumab-induced Th2 cell activation. As shown in Fig. 10a, Ipilimumab treatment significantly enhanced the in vitro production of Th2-type cytokines, including IL-4, IL-6 and IL-10. This was abrogated by treatment with anti-B7 mAbs in vivo. To test the impact of anti-B7 mAbs on de novo priming of CD8 T cells in peripheral lymphoid organs, we immunized tumor-bearing mice with SIY peptide and treated the mice with Ipilimumab in the presence or absence of anti-B7 mAbs. Representative profiles of SIY-H-2K^b^-specific T cells are shown in Fig. 10b, while summary data from a representative study are presented in Fig. 10c. In the absence of anti-B7 mAbs, immunization with SIY peptide induced significant expansion of SIY-specific T cells (Fig. 10b, c). This appears to be slightly increased by Ipilimumab treatment. In the presence of anti-B7 mAbs, however, no de novo priming of SIY-specific T cells was observed. Since anti-B7 mAbs did not interfere with immunotherapeutic effect of Ipilimumab (Fig. 9), the findings presented in Fig. 10 suggest that *de novo*T cell priming after Ipilimumab treatment is not required for achieving the immunotherapeutic effects of Ipilimumab.

### Blocking the B7-Ctla-4 interaction is not associated with immunotherapeutic effect of anti-mouse Ctla-4 mAbs

The concept that CTLA-4 is a cell-intrinsic negative regulator of T cell function arose from the stimulatory effect of both intact and Fab of two anti-mouse Ctla-4 mAbs,^35,36^ 4F10 and 9H10, even though no data were presented to demonstrate that these antibodies block the B7-Ctla-4 interaction. More recently, a third anti-mouse Ctla-4 mAb, 9D9, was reported to have therapeutic effect in tumor-bearing mice and cause local depletion of Treg cells in tumor microenvironment.^10^ We therefore set out to test all three commercially available anti-mouse Ctla-4 mAbs that had been shown to induce tumor rejection for their ability to block the B7-Ctla-4 interaction under physiologically relevant conditions. As a first test, we used increasing amounts of anti-mouse Ctla-4 mAbs (up to a 2000-fold molar excess over Ctla-4-Fc) to block binding of biotinylated Ctla-4-Fc to plates coated with mB7-1 and mB7-2. This revealed that anti-mouse Ctla-4 mAb 9H10 did not block the the mB7-1-Ctla-4 interaction even at the highest concentration tested, although a modest blocking was observed when 9D9 was used at very high concentrations (Supplementary information, Figure S9A). Whereas mAb 9D9 effectively blocked the mB7-2-Ctla-4 interaction, 9H10 failed to do so (Supplementary information, Figure S9B). Interestingly, while 9D9 showed strong binding to soluble Ctla-4Fc, 9H10 showed poor binding (Supplementary information, Figure S9C), even though it was more potent than 9D9 in binding-immobilized mouse Ctla-4-Fc (Supplementary information, Figure S9D). Since lack of any blocking activity by 9H10 in this assay may simply reflect its poor binding to soluble Ctla-4-Fc, we again used upregulation of B7-1 and B7-2 on DCs in WT mice (*Ctla4*^*m/m*^) to measure blocking of the B7-Ctla-4 interaction in vivo. This did not upregulate B7-1 expression on DCs, while 9D9 increased mB7-1 level by 15% (*P* < 0.05) (Supplementary information, Figure S9E, F, 9H10). Interestingly, whereas 9D9 clearly upregulated mB7-2 on DCs, 9H10 failed to do so. Therefore, 9H10, the first and most extensively studied tumor immunotherapeutic anti-Ctla-4 mAb does not block the B7-Ctla-4 interactions. These data argue against a role for blocking the B7-Ctla-4 interaction in the induction of antitumor immunity by anti-mouse Ctla-4 mAbs. Since both mAbs showed comparable immunotherapeutic effect and comparable depletion of Treg cells in the tumor microenvironment,^10^ local depletion of Treg cells, rather than blockade of the mB7-CTLA-4 interaction, provides a unifying explanation for the therapeutic effect of anti-mouse CTLA-4 mAbs. Interestingly, whereas 4F10 blocked the B7-Ctla-4 interaction in vitro, it failed to induce upregulation of B7 on DCs in vivo (Supplementary information, Figure S10).

## Discussion

Although Ipilimumab was called a blocking mAb based on the fact that it blocks the B7-CTLA-4 interaction when B7 is added in soluble form, our data have demonstrated that it barely blocks B7-CTLA-4 interaction under physiologically relevant conditions, including when B7-1 and B7-2 were immobilized to solid phase or expressed on the cell membrane; when the B7-CTLA-4 complex was formed prior to exposure to anti-CTLA-4 mAbs; when both B7 and CTLA-4 were expressed as cell surface molecules; and particularly when B7 and CTLA-4 were presented as naturally expressed on DC and T cells, respectively, and when animals receive antibody treatment in vivo. More importantly, Ipilimumab confers its immunotherapeutic effect without blocking the B7-CTLA-4 interaction because it remains effective either when at least 50% of CTLA-4 does not bind to the antibody in *Ctla4* ^*h/m*^ mice or when host B7 is masked by anti-B7 mAbs.

A surprising finding in our study is the marked difference in Ipilimumab-blocking activity depending on whether B7 or CTLA-4 proteins are in soluble phase. This can now be explained by two pieces of data. First, Ipilimumab does not break up existing B7-CTLA-4 complexes. Second, the on-rate for soluble CTLA-4 binding to plate-bound B7 is at least three times faster than that of soluble B7 binding to plate-bound CTLA-4. Together, these data suggest that when B7 is added in solution, Ipilimumab has more chance to bind to free CTLA-4 than when B7 is immobilized and has more chance to block the CTLA-4-B7 interaction before the complex is formed. Since the CTLA-4-antibody interaction is dynamic, the CTLA-4 molecules that disassociate from antibody can bind to immobilized B7 and become “immune” to blocking by Ipilimumab. As such, a partial overlap between B7-binding and Ipilimumab-binding sites, on CTLA-4, as recently reported,^37^ does not necessarily enable blocking of the B7-CTLA-4 interaction under physiologically relevant conditions.

The differential activity between L3D10 and Ipilimumab to break pre-formed complex remains to be elucidated. While *K*_on_ of Ipilimumab (2.6 × 10^5^/Ms or 3.83 × 10^5^/Ms)^14,16^ is lower than that of soluble B7 (1–4 × 10^6^/Ms, this study), L3D10 does not have a faster *K*_on_ (2.07 × 10^5^/Ms) than Ipilimumab.^15^ Therefore, the *K*_on_ or *K*_off_ value does not offer an explanation for the ability of the two antibodies to differentially block the B7-CTLA-4 interaction when B7 is immobilized. A more plausible explanation is that once the complex is formed, the CTLA-4 conformation is changed in such a way as to prevent Ipilimumab from binding. The published data on the Ipilimumab-CTLA-4 complex show a partial overlap between the Ipilimumab epitope and the B7-binding site on CTLA-4^37^ that is consistent with this explanation.

To model the physiological conditions under which both B7 and CTLA-4 are present on the cell surface, we performed a trans-endocytosis assay using CHO cells expressing either GFP-tagged B7-1, B7-2 or OFP-tagged CTLA-4. To overcome the complication of associated signaling through the cross-linking of CTLA-4, it is important to use the Fab fragment rather than bivalent antibodies. Our data clearly demonstrate that despite robust binding of the Ipilimumab Fab fragment (at a concentration 10-fold greater than needed for saturated binding −10 μg/ml) to cell surface CTLA-4, there was only 15−30% inhibition of trans-endocytosis of B7-1 and B7-2. More importantly, by molar ratio, this concentration would translate to around a 50% higher concentration than the plasma concentration achieved by clinically effective dosing. Similarly, when cell surface CTLA-4 is stabilized by the Y201V mutation to allow a stable B7-CTLA-4-mediated cell–cell interaction, high doses of Ipilimumab Fab only result in <20% inhibition. Since the clinical effective dosing is inadequate to cause effective inhibition of either B7 trans-endocytosis or cell surface interaction mediated by B7 and CTLA-4, our cell-based in vitro assays strongly argue against a CTLA-4 blockade as the mechanism of action for the clinically effective drug.

The predictions from these in vitro studies are validated by the in vivo studies. Our in vivo assay is based on the recent discovery that CTLA-4 functions by causing downregulation of B7 on DCs via trans-endocytosis.^12,27^ Because of this unique property, one would not expect stable DC–Treg cell conjugation mediated by B7-CTLA-4 interactions in vivo. Rather, blocking CTLA-4-mediated trans-endocytosis directly results in higher expression of B7 on DCs.^12,27^ To rule out a potential caveat that upregulation of B7 is due to signaling by anti-CTLA-4 mAbs, we used heterozygous mice with both mouse and human *CTLA4* alleles.^38^ In this model, anti-human CTLA-4 mAbs can be an effective agonist but not antagonist because it will not be able to bind >50% of CTLA-4 molecules. The fact that the blocking anti-CTLA-4 mAb L3D10 induces B7 upregulation in the homozygous but not heterozygous mice confirmed the specificity of the in vivo assay and showed that functional blocking would need to block more than 50% of CTLA-4, perhaps because trans-endocytosis can be accomplished with 50% CTLA-4 being unoccupied. As such, upregulation of B7 on DCs represents the most physiologically relevant and direct readout for blockade of the B7-CTLA-4 interaction.

The lack of contribution from a B7-CTLA-4 blockade is also demonstrated by the absence of a correlation between blocking and therapeutic efficacy. Despite more than 1000-fold differences in blocking the B7-CTLA-4 interaction, L3D10 and Ipilimumab are comparable in inducing tumor rejection. Therefore, such a blockade does not significantly contribute to the efficacy of the anti-CTLA-4 mAbs. Interestingly, since L3D10 efficiently induces tumor rejection in heterozygous mice in which it cannot functionally block all the B7-CTLA-4 interaction, such a  blockade is not necessary for tumor rejection even for an antibody able to achieve blocking. Remarkably, humanized L3D10 progeny that have lost blocking activities remain fully active in immunotherapy. These data refute the hypothesis that anti-CTLA-4 mAbs operate primarily through checkpoint blockade.^1^ By refuting the prevailing hypothesis, our data suggest that improving the blocking activities of the anti-CTLA-4 mAbs is unlikely to be the correct approach to increase the therapeutic efficacy of anti-CTLA-4 mAbs. Our companion paper further validated this concept.

A small proportion of human subjects are known to express soluble B7-1.^39^ Since Ipilimumab blocks the interaction between soluble CD80 and CTLA-4, it is of interest to consider whether blocking soluble CD80 may be responsible for tumor rejection. We consider this unlikely for two reasons. First, since soluble CD80 is known to promote tumor rejection as it provides co-stimulation for T cells,^40^ blocking this interaction should suppress rather than promote tumor rejection. Second, the humanized L3D10 clones HL12 and HL32, which have lost the ability to block the B7-CTLA-4 interaction regardless of whether CD80 is immobilized or in soluble form, are potent inducers of tumor rejection.

Meanwhile, our in vivo studies showed that all therapeutically effective anti-CTLA-4 antibodies used herein are remarkably effective in causing local Treg depletion. Our data provide clear evidence that, much like anti-mouse Ctla-4 mAbs, anti-human CTLA-4 mAbs, including the clinically effective Ipilimumab, may have provided therapeutic effect through ADCC. This hypothesis is verified by a critical role for host FcR in Ipilimumab-induced tumor rejection. Our work supports the hypothesis that local depletion of Treg cells within the tumor environment is the main mechanism for clinically effective anti-human CTLA-4 mAb, and hence suggests new approaches to develop the next generation of anti-CTLA-4 mAb for cancer immunotherapy by selectively enhancing local Treg depletion regardless of blocking activity.

The requirement for induction of local Treg depletion within the tumor microenvironment to achieve therapeutic effects is inconsistent with another postulate of the checkpoint blockade hypothesis,^1^ which states that unlike anti-PD-1/PD-L1 antibodies, anti-CTLA-4 antibodies promote tumor rejection by preventing negative signaling in the peripheral lymphoid organs. By showing that B7 blockade prevented de novo T cell activation without affecting therapeutic effect of Ipilimumab, our data refute this postulate. Importantly, instead of contributing to tumor rejection, our companion paper demonstrates that systemic T cell activation strongly correlates with an immunotherapy-related adverse effect.

Finally, accumulating genetic data in mice suggest that the original concept^35,36^ that CTLA-4 negatively regulates T cell activation and that such regulation is achieved through Shp-2^41,42^ may need to be revisited.^43^ Thus, while the severe autoimmune diseases in *Ctla4*^*−/−*^ mice have been used to support the notion of CTLA-4 as a cell-intrinsic negative regulator for T cell activation,^44,45^ at least three lines of genetic data have since emerged that are not consistent with this view. First, lineage-specific deletion of the *Ctla4* gene in Treg cells but not in effector T cells is sufficient to recapitulate the autoimmune phenotype observed in mice with a germline deletion of the *Ctla4* gene,^26^ although the onset of fatality is slower than in mice with either germline or pan-T cell deletion of the gene.^44–46^ While the function of *Ctla4* in Foxp3^−^ cells remains to be investigated, these data suggest that development of fatal autoimmunity in the *Ctla4*^*−/−*^ mice does not require deletion of *Ctla4* in effector T cells. Second, in chimeric mice having both WT and *Ctla4*^*−/−*^ T cells, the autoimmune phenotype was prevented by the co-existence of WT T cells.^47^ These data again strongly argue that autoimmune diseases were not caused by lack of cell-intrinsic negative regulator. The lack of a cell-intrinsic negative regulator effect is also demonstrated by the fact that in the chimeric mice, no preferential expansion of *Ctla4*^*−/−*^ T cells was observed during viral infection.^48^ Third, T cell-specific deletion of *Shp2*, which was proposed to mediate negative regulation of CTLA-4,^41,42^ turned out to reduce rather than enhance T cell activation.^49^ In the context of these genetic data reported since CTLA-4 was proposed as negative regulator for T cell activation, our present data call for a reappraisal of the CTLA-4 checkpoint blockade hypothesis in cancer immunotherapy.

## Materials and methods

### Animals

*CTLA4*-humanized mice that express the CTLA-4 protein with 100% identify to human CTLA-4 protein under the control of endogenous mouse *Ctla4* locus have been previously described.^38^ Homozygous knock-in mice (*Ctla4*^*h/h*^) were backcrossed into the C57BL/6 background for at least 10 generations. Heterozygous mice (*Ctla4*^*h/m*^) were produced by crossing the *Ctla4*^*h/h*^ mice with wild type (WT) BALB/c or C57BL/6 mice. WT C57BL/6 mice were purchased from Charles River Laboratories. Human cord blood CD34^+^ stem cell-reconstituted NSG™ mice were obtained from the Jackson Laboratories (Bar Harbor, Maine). All animals (both female and male, 6-16 weeks old and age-matched in each experiment) were included in the analysis. No blinding or randomization was used, except that mice were randomly assigned to each group. All mice were maintained at the Research Animal Facility of Children’s Research Institute at the Children’s National Medical Center. All studies involving mice have been approved by the Institutional Animal Care and Use Committee.

### Cell culture

No cell lines used in this study were listed in the database of cross-contaminated or misidentified cell lines suggested by International Cell Line Authentication Committee (ICLAC). CHO cells and L929 cells transfected with mouse or human B7-1 or B7-2 have been described previously.^20,29^ B7-1-transfected J558 cells^22^ and P815 cells transfected with B7-H2-GFP^50^ have been described previously. The murine colon tumor cell line MC38 was also described previously.^5^ The melanoma cell line B16-F10 (ATCC® CRL-6475™) and HEK293T cells (ATCC® CRL-11268™) were originally purchased from ATCC (Manassas, VA, USA). After receiving from the vendors, cell passages were kept minimal before in vivo testing. All cell lines were incubated at 37 °C and were maintained in an atmosphere containing 5% CO_2_. Cells were grown in DMEM (Dulbecco’s Modified Eagle Medium, Gibco) supplemented with 10% FBS (Hyclone), 100 units/ml of penicillin and 100 μg/ml of streptomycin (Gibco).

### Antibodies

Mouse anti-human CTLA-4 mAb L3D10 has been described.^15^ The anti-CTLA-4 mAb L3D10 used in the study was a chimeric antibody consisting of human IgG1-Fc and the variable regions of L3D10. Recombinant WT (M1) and mutated (M17, M17-4) hCTLA-4 proteins, as well as recombinant antibodies including parental and fully humanized L3D10 (clones HL12 and HL32) were produced by Lakepharma, Inc (Belmont, CA, USA). Recombinant Ipilimumab with amino acid sequence disclosed in WC500109302 and http://www.drugbank.ca/drugs/DB06186 was provided by Alphamab Inc. (Suzhou, Jiangsu, China), or Lakepharma Inc (San Francisco, CA, USA). Clinical Ipilimumab was also used for much of the studies (Figs. 1d, 2a, b, 5, 6 and 7b right panel, 7d–j, 9, Supplementary information, Figures S2A and S7). Human IgG-Fc (No azide) was bulk ordered from Athens Research and Technology (Athens, GA, USA). Anti-mouse CD16/32 mAb 2.4G2, anti-mouse B7-1mAb 1G10, anti-mouse B7-2 mAb GL1, anti-mouse Ctla-4 mAbs 9D9 and 9H10, control hamster IgG, control mouse IgG2b MPC-11 and human CTLA-4-Fc were purchased from Bio-X-Cell Inc. (West Lebanon, NH, USA). Purified hamster anti-mouse Ctla-4 mAb 4F10 was purchased from BD Biosciences (San Jose, CA, USA). Purified and biotinylated hamster IgG isotype control antibodies used for in vitro blocking assays were purchased from eBioscience (San Diego, CA, USA). Fusion proteins for human B7-1-Fc, B7-2-Fc and polyhistidine-tagged human CTLA-4 were purchased from Sino Biological Inc. (Beijing, China). Recombinant mouse Ctla-4-Fc protein was purchased from BioLegend (San Diego, CA, USA). Biotinylation was completed by conjugating EZ-Link Sulfo-NHS-LC-Biotin (Thermo Scientific) to desired proteins according to the manufacturer’s instructions. Alexa Fluor 488-conjugated goat anti-human IgG (H+L) cross-adsorbed secondary antibody was purchased from ThermoFisher Scientific, USA. The levels of cytokines IL-4, IL-6 and IL-10 were evaluated by Cytometric Beads Array (BD Biosciences, Catalogue number 560485) following the manufacture’s protocol. SIY peptide was purchased from MBL International Corporation (Woburn, MA, USA), and SIY-specific CD8 T cells were detected by H-2K^b^ tetramer SIYRYYGL-PE (MBL Code # TS-M008-1). H-2K^b^ tetramer OVA (SIINFEKL)-PE provided by NIH (#31074) was used as negative control for flow stainings.

### Assays for in vitro and in vivo blockade of B7-CTLA-4 interaction

Three assays were employed to assess the blocking activities of anti-CTLA-4 mAbs. First, we coated plates with either CTLA-4-Fc or their ligand, B7-1. Biotinylated fusion proteins were used in soluble phase in the binding assay, with the amounts of protein bound measured by horse-radish peroxidase (HRP)-conjugated avidin (Pierce High Sensitivity NeutrAvidin-HRP, Thermo Scientific Inc.). Proteins were coated in bicarbonate buffer (0.1M) at 4 °C and the binding assays were performed at room temperature.

Second, we used flow cytometry to detect binding of biotinylated fusion protein to CHO cells transfected to express mouse or human B7-1 and B7-2 on the cell surface. In each assay consisting of 105 μl PBS solution, 1.2 × 10^5^ CHO cells were incubated with 200 ng biotinylated human or mouse CTLA-4 protein, along with varying doses of anti-human or mouse CTLA-4 mAbs or control IgG, for 30 min at room temperature. The amounts of bound receptors were measured using phycoethrythorin (PE)-conjugated streptavidin purchased from BioLegend (San Diego, CA, USA). Flow cytometry was performed using FACS CantoII (BD Biosciences), and data were analyzed by FlowJo (Tree Star Inc.).

Third, we used the upregulation of B7-1 and B7-2 by anti-CTLA-4 mAbs as the readout for blockade of the B7-1-CTLA-4 and the B7-2-CTLA-4 interaction. Briefly, age and gender-matched mice received 500 μg of antibodies or their controls intraperitoneally. At 24 h after injection, mice were killed and their spleen cells were stained with antibodies against CD11c (clone N418), CD11b (clone M1/70), B7-1 (clone 16-10-A1) and B7-2 (clone PO3.1) and isotype control Abs purchased from eBioscience Inc. (San Jose, CA, USA). NSG^TM^ mice reconstituted with human CD34^+^ cord blood cells received the same doses of antibodies. The spleens were meshed between two frosted microscope slides, and then incubated for 20 min at 37 °C in 5 ml buffer containing 100 μg/ml Collagenase Type IV and 5 U/ml DNase I. A cell suspension was prepared by gently pushing the digested nodes through a cell strainer, and stained with the antibodies specific for the following markers: hB7-1, clone 2D10 (Biolegend Cat. No 305208); hB7-2: clone IT2.2 (BioLegend, Cat No. 305438); hCD11c, clone 3.9; BioLegend Cat No. 301614); HLA-DR, clone L243 (BioLegend Cat. No. 307616); hCD45, clone HI30 (BioLegend, Cat. No. 304029).

### Trans-endocytosis assay and cell–cell interaction assay

Plasmids with GFP (C-GFPSpark tag)-tagged human B7-2/B7-1 and OFP (C-OFPSpark tag)-tagged human CTLA-4 cDNA were purchased from Sino Biological Inc. (Beijing, China) and used to establish stable CHO cell lines expressing either molecule. To measure inhibition of trans-endocytosis by anti-CTLA-4 mAbs, the Fab fragments were prepared with the Pierce™ Fab Preparation Kit (Thermo Scientific, USA) following the manufacturer’s instruction. Given doses of the Fab or control hIgG-Fc proteins were added to GFP-tagged B7-2-expressing CHO cells immediately prior to their co-culturing with OFP-tagged CTLA-4-expressing CHO cells at 37 °C for 4 h.

Plasmids encoding OFP-tagged human CTLA-4 or human *CTLA4*^*Y201V*^ cDNA was used to establish stable HEK293T cell lines. After overnight suspension culture in 15 ml centrifuge tubes, B7-GFP-tagged CHO cells and *CTLA4*^*Y201V*^-OFP-tagged HEK293T cells were co-incubated at an approximate 2:1 ratio at 4 °C for 2 h. Given doses of the Fab or control hIgG-Fc proteins were added to the mixed cells immediately prior to their co-culturing. For both trans-endocytosis and cell–cell interaction assays, 1 × 10^5^ B7-GFP-tagged CHO cells were used in each single test. Trans-endocytosis and cell–cell interactions were determined by flow cytometry based on acquisition of GFP signal from the B7-GFP-transfected CHO cells by CTLA-4-OFP-transfected CHO cells or *CTLA4*^*Y201V*^-OFP-transfected HEK293T cells.

The following formula was used for the calculation of both assays:

% trans-endocytosis or cell–cell interaction = (GFP^+^OFP^+^%)/(GFP^+^OFP^+^%+ GFP^−^OFP^+^%)

### Kinetics of B7-CTLA-4 interaction

Binding experiments were performed on Octet Red96 at 25 °C by Lakepharma Inc. Biotinylated B7-1-Fc or CTLA-4-Fc were captured on Streptavidin (SA) biosensors. Loaded biosensors were then dipped into a dilution of either B7-1-Fc or CTLA-4-Fc at variable concentrations (300 nM start, 1:3 down, seven points). The association rate constant, *K*_a_, describes the number of B7-1-CTLA-4 complexes formed per second in a 1 M solution of CTLA-4-Fc or B7-1-Fc.

### Impact of anti-CTLA-4 mAb on pre-formed B7-CTLA-4 complex

For ELISA experiments, hB7-1-Fc or hB7-2-Fc were pre-coated on 96-well high-binding polystyrene plates at given concentrations in coating buffer overnight. After washing away the unbound protein, the plates were blocked with 1% BSA in PBST and then incubated with 0.25 μg/ml biotinylated CTLA-4-Fc protein for 2 h. After washing away the unbound protein, given doses of hIgG-Fc/Ipilimumab/L3D10 were added and incubated for 2 h. The plate-bound biotinylated CTLA-4-Fc was detected with HRP-conjugated streptavidin. For flow cytometric assays, surface hB7-1 or mB7-2-expressing CHO cells (1×10^5^/test) were incubated with soluble biotinylated CTLA-4-Fc (200 ng/test) for 30 min at room temperature. After washing, cells were incubated in 100 μl DPBS buffer for the indicated minutes along with the intended doses of control hIgG-Fc or anti-CTLA-4 mAbs. The amounts of B7-bound CTLA-4-Fc were detected with PE-streptavidin by flow cytometry, and the mean fluorescence intensity of PE was calculated from triplicated samples.

### Tumor growth and regression assay

Mice with either heterozygous or homozygous knock-in of the human *CTLA4* gene were challenged with given numbers of either colorectal cancer cell MC38 or melanoma cell line B16-F10. Immunotherapies were initiated at 2, 7 or 11 days after injection of tumor cells with indicated doses. The tumor growth and regression were determined by tumor volume as the readouts. The volumes (*V*) were calculated using the following formula:

*V* = *ab*^2^/2, where *a* is the long diameter, while *b* is the short diameter.

### Biostatistics

The specific tests used to analyze each set of experiments are indicated in the figure legends. For each statistical analysis, appropriate tests were selected on the basis of whether the data were normally distributed by using the Shapiro–Wilk test. Data were analyzed using an unpaired two-tailed Student’s *t* test or Mann–Whitney test to compare between two groups, either one-way or two-way ANOVA (analysis of variance) with Sidak’s correction for multiple comparisons, two-way repeated measures ANOVA for behavioral tests. Sample sizes were chosen with adequate statistical power on the basis of the literature and past experience. No samples were excluded from the analysis, and experiments were not randomized except what was specified. Blinding was not carried out during animal group allocation but was for some measurements made in the study (i.e., tumor size measuring, flow cytometrical assay of B7 expression). In the graphs, *y*-axis error bars represent S.E.M. or S.D. as indicated. Statistical calculations were performed using Excel (Microsoft), GraphPad Prism software (GraphPad Software, San Diego, California) or R Software (https://www.r-project.org/).

### Data availability

All data generated or analyzed during this study are included in this published article and its supplementary information files. No data sets were generated or analyzed during the current study.

## Electronic supplementary material

Supplementary information, Table S1

Supplementary information, Figure S1

Supplementary information, Figure S2

Supplementary information, Figure S3

Supplementary information, Figure S4

Supplementary information, Figure S5

Supplementary information, Figure S6

Supplementary information, Figure S7

Supplementary information, Figure S8

Supplementary information, Figure S9

Supplementary information, Figure S10
