# Supplementary material for: A reappraisal of CTLA-4 checkpoint blockade in cancer immunotherapy
Source: Cell Res. 2018 Feb 22;28(4):416–32. doi: 10.1038/s41422-018-0011-0 (PMC5939050; doi:10.1038/s41422-018-0011-0)
Supplement: Supplementary file 1 — Supplementary information, Table S1 [file 41422_2018_11_MOESM1_ESM.pdf]

Supplementary information, Table S1

Binding characteristics of humanized L3D10 clones used in this study.

Antibodies	Antigen	KD (M)	K_{on}(1/Ms)	K_{dis}(1/s)
HL12	CTLA4Fc	7.2E-09	2.3E+05	1.6E-03
HL32	CTLA4Fc	7.1E-09	2.7E+05	1.9E-03
L3D10	CTLA4Fc	2.3E-09	3.5E+05	8.0E-04

Multi-concentration kinetic experiments were performed on the Octet Red96 system (ForteBio). Anti-hIgG-Fc biosensors (ForteBio, #18-5064) were hydrated in sample diluent (0.1% BSA in PBS and 0.02% Tween 20) and preconditioned in pH 1.7 Glycine. The antigen was diluted using a 7-point, 2-fold serial dilution starting at 600 nM with sample diluent. All antibodies were diluted to 10 µg/ml with sample diluent and then immobilized onto anti-hIgG-Fc biosensors for 120 seconds. After baselines were established for 60 seconds in sample diluent, the biosensors were moved to wells containing the antigen at a series of concentrations to measure the association. Association was observed for 120 seconds and dissociation was observed for 180 seconds for each protein of interest in the sample diluent. K_{on}, on rate; K_{dis}, off rate; KD, the equilibrium dissociation constant.
