# Supplementary material for: A reappraisal of CTLA-4 checkpoint blockade in cancer immunotherapy
Source: Cell Res. 2018 Feb 22;28(4):416–32. doi: 10.1038/s41422-018-0011-0 (PMC5939050; doi:10.1038/s41422-018-0011-0)
Supplement: Supplementary file 2 — Supplementary information, Figure S1 [file 41422_2018_11_MOESM2_ESM.pdf]

Figure S1

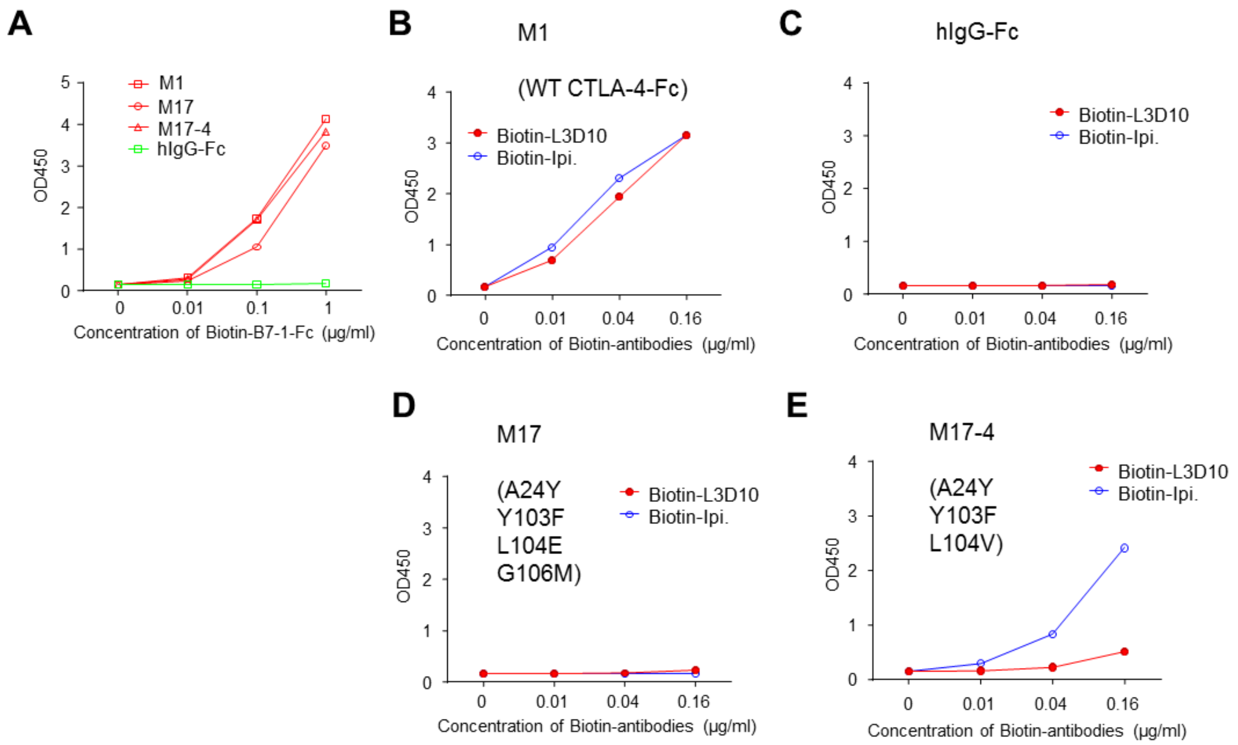

Supplementary information, Figure S1 Mutational analysis of CTLA-4-Fc reveals that Ipilimumab and L3D10 bind to distinct but overlapping epitope. **(A-E)** Based on the crystal structure and variation of mouse and human CTLA-4 sequences, we generated hCTLA-4 mutants M17 and M17-4. **(A)** The integrity of CTLA-4 molecules was confirmed by their ability to bind to biotinylated B7-1. Control hIgG-Fc, WT (M1) and mutated (M17 and M17-4) hCTLA-4 proteins were coated on 96-well plate at a concentration of 1μg/ml. Varying doses of biotinylated hB7-1-Fc were added to test their binding abilities, which were measured by streptavidin-HRP. **(B-E)** Control hIgG-Fc, WT (M1) and mutated (M17 and M17-4) hCTLA-4 proteins were coated on 96-well plate at a concentration of 1μg/ml. Varying doses of biotinylated L3D10 or Ipilimumab were added to test their binding abilities to hCTLA-4 molecules. The specificity of the binding is

confirmed by their binding to WT CTLA-4-Fc (**B**) but not hIgG-Fc (**C**). While 4 mutations in M17 completely inactivated the binding to both L3D10 and Ipilimumab (**D**), 3 mutations in M17-4 drastically abrogated the binding to L3D10 but not Ipilimumab (**E**).
