# Supplementary material for: A reappraisal of CTLA-4 checkpoint blockade in cancer immunotherapy
Source: Cell Res. 2018 Feb 22;28(4):416–32. doi: 10.1038/s41422-018-0011-0 (PMC5939050; doi:10.1038/s41422-018-0011-0)
Supplement: Supplementary file 3 — Supplementary information, Figure S2 [file 41422_2018_11_MOESM3_ESM.pdf]

Figure S2

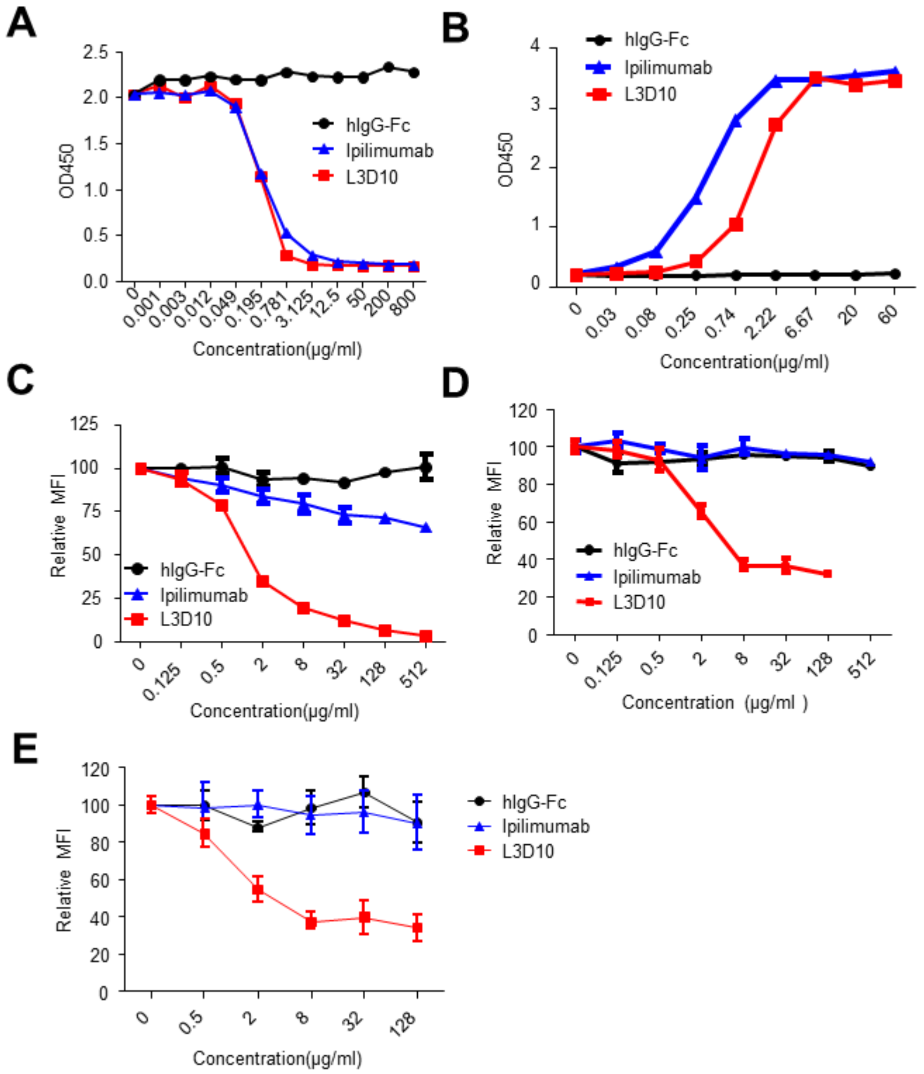

Supplementary information, Figure S2 Ipilimumab exhibits poor blocking activity for B7-CTLA-4 interactions if B7 is immobilized. **(A)** Both Ipilimumab and L3D10 potently block B7-CTLA-4 interaction if soluble B7-1 is used for the binding assay. Varying doses of anti-human CTLA-4 mAbs were added along with 0.025 μg/ml of biotinylated human B7-1-Fc to plate coated with 1μg/ml human CTLA-4-Fc. The amounts of B7-1-Fc bound to plates were measured using HRP-conjugated avidin. Data shown are means of duplicates and are representative of two independent experiments. **(B)** Ipilimumab

binds better than L3D10 to biotinylated human CTLA-4-Fc. Varying doses of anti-human CTLA-4 mAbs or control IgG were coated onto the plate. Biotinylated CTLA4-Fc was added at 0.25 μg/ml. The amounts of CTLA-4 bound to plates were measured using HRP-conjugated streptavidin. Data shown are means of duplicates and are representative of two independent experiments. **(C)** Detectable but modest blocking of mouse B7-1-human CTLA-4 interaction by Ipilimumab when mB7-1 is expressed on CHO cells. Varying doses of anti-human CTLA-4 mAbs were added along with 200ng of human CTLA-4-Fc to 1.2 X 10^5 CHO cells expressing mouse B7-1. Data shown are means and S.D. of triplicate data and are representative of three independent experiments. **(D)** L3D10 but not Ipilimumab blocks interaction between polyhistindine tagged human CTLA-4 and CHO cells expressing human B7-1. 1.2 X10^5 CHO cells expressing human B7-1 were incubated with 200ng biotinylated and polyhistidine-tagged CTLA-4 along with given doses of antibodies. The amounts of CTLA-4-Fc bound to CHO cells were detected with PE-streptavidin by flow cytometry. Data (Mean ± S.D.) shown are normalized mean fluorescence intensity (MFI) of triplicate samples and are representative of two independent experiments. **(E)** Ipilimumab and L3D10 exhibited differential blocking activity for the interaction between soluble hCTLA-4 and cell surface expressed hB7-1. hB7-1-positive, FcR-negative L929 cells (1X10^5/test) were incubated with biotinylated CTLA-4-Fc (200ng/test) along with given doses of antibodies. The amounts of B7-bound CTLA-4-Fc were detected with PE-streptavidin, and mean fluorescence intensity (MFI) of PE was calculated. Data represent the results of two independent experiments.
