# Supplementary material for: A reappraisal of CTLA-4 checkpoint blockade in cancer immunotherapy
Source: Cell Res. 2018 Feb 22;28(4):416–32. doi: 10.1038/s41422-018-0011-0 (PMC5939050; doi:10.1038/s41422-018-0011-0)
Supplement: Supplementary file 4 — Supplementary information, Figure S3 [file 41422_2018_11_MOESM4_ESM.pdf]

Figure S3

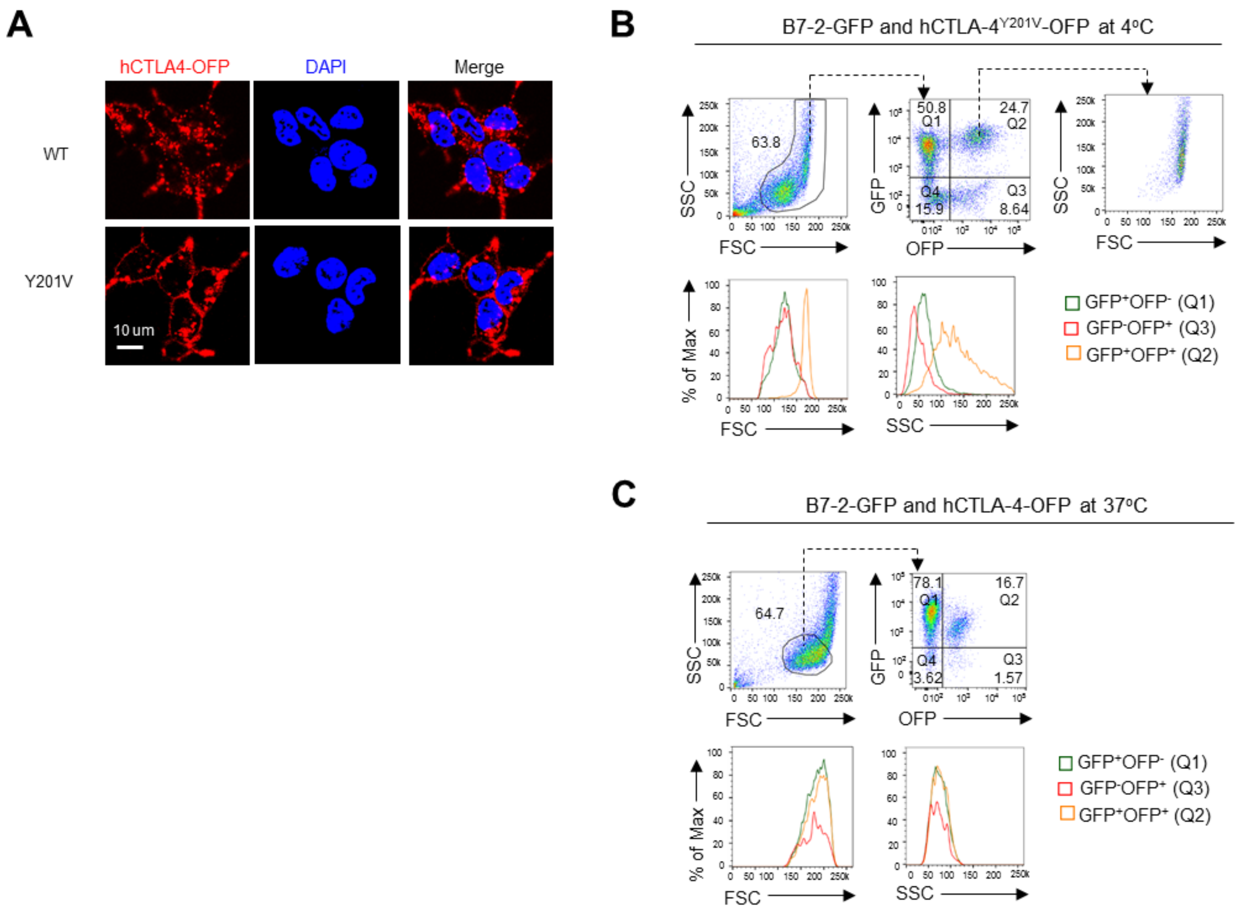

Supplementary information, Figure S3 Characterization of cellular assays for B7-CTLA-4 interaction. **(A)** Confocal images of 293T cells stably expressing wild-type (WT, top panels) and Y201V mutant (bottom panels) of human hCTLA-4-OFP proteins. Note that while WT hCTLA-4 is predominantly intracellular, mutant hCTLA-4 molecules show a clear pattern of plasma membrane distribution. **(B)** $GFP^{+}OFP^{+}$ cells in cell-cell binding assays used in Figure 3 are cell-cell aggregates based on their forward and side scatters. Representative flow profiles of hB7-2-GFP-CHO and hCTLA-4^{Y201V}-OFP-293T cells co-incubated at 4°C for 2h. Top panels show forward vs. side scatters of the $GFP^{+}OFP^{+}$ cells, while the lower panels show comparisons of the forward scatters (left) and side scatters (right) of single vs. double positive cells. **(C)** Characterization of the

transendocytosis assay. The top panels show the gating used for data presented in Figure 4, while the lower panels show that after co-incubation at 37°C for 4 hours, CTLA-4-OFP-CHO cells acquired GFP signals from hB7-2-GFP-CHO cells without alteration in the forward and side scatters.
