# Supplementary material for: A reappraisal of CTLA-4 checkpoint blockade in cancer immunotherapy
Source: Cell Res. 2018 Feb 22;28(4):416–32. doi: 10.1038/s41422-018-0011-0 (PMC5939050; doi:10.1038/s41422-018-0011-0)
Supplement: Supplementary file 5 — Supplementary information, Figure S4 [file 41422_2018_11_MOESM5_ESM.pdf]

Figure S4

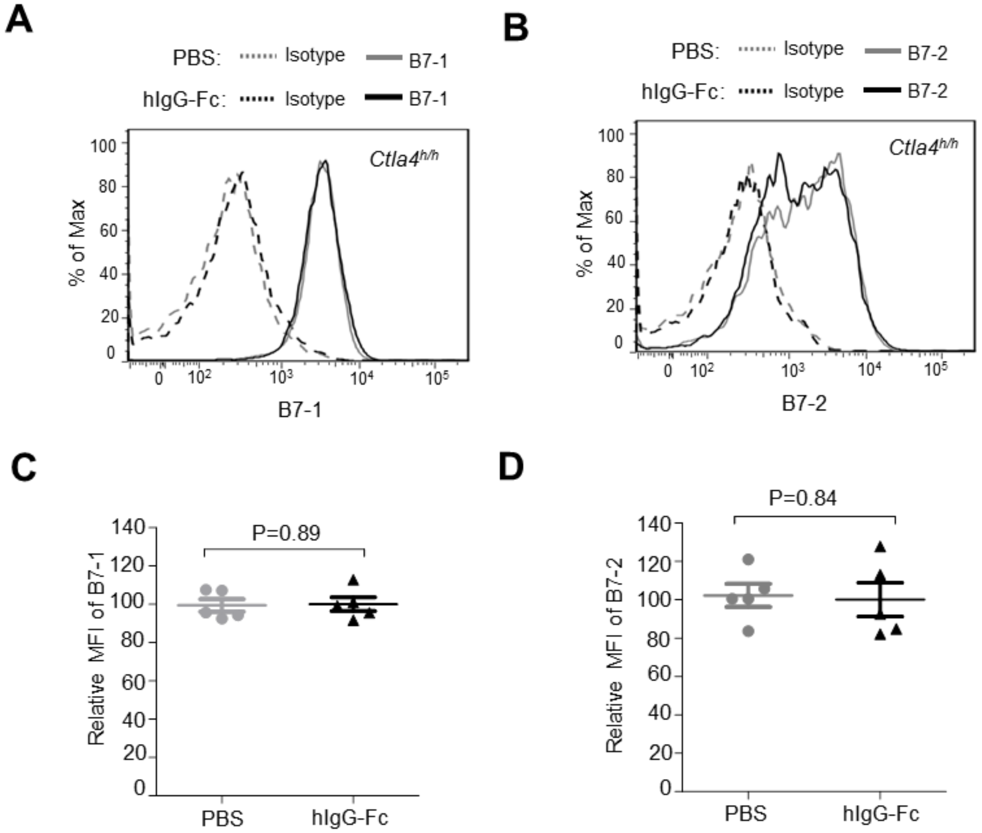

Supplementary information, Figure S4 Despite somewhat higher levels of endotoxin detected in the hIgG-Fc preparation than the antibody preparations, hIgG-Fc did not up-regulate B7-1 and B7-2 expressions on mouse spleen DCs. **(A, B)** Representative profiles of B7-1 (**A**) and B7-2 (**B**) expression among the spleen DCs gated as depicted in Figure 5B from *Ctla4*$^{h/h}$ mice treated with 500µg of hIgG-Fc or equal volume of PBS. **(C, D)**Summarization of mean fluorescence intensities for B7-1 (**C**) and B7-2 (**D**) expressed on spleen DCs. n=5 *Ctla4*$^{h/h}$ mice for each group. Therefore, the profiles of the control hIgG-Fc-treated mice reflect the basal expression levels of B7-1 and B7-2. Thus, the lack of effect of Ipilimumab over hIgG-Fc indicates its inability to up-regulate B7-1 and B7-2 *in vivo*.
