# Supplementary material for: A reappraisal of CTLA-4 checkpoint blockade in cancer immunotherapy
Source: Cell Res. 2018 Feb 22;28(4):416–32. doi: 10.1038/s41422-018-0011-0 (PMC5939050; doi:10.1038/s41422-018-0011-0)
Supplement: Supplementary file 6 — Supplementary information, Figure S5 [file 41422_2018_11_MOESM6_ESM.pdf]

Figure S5

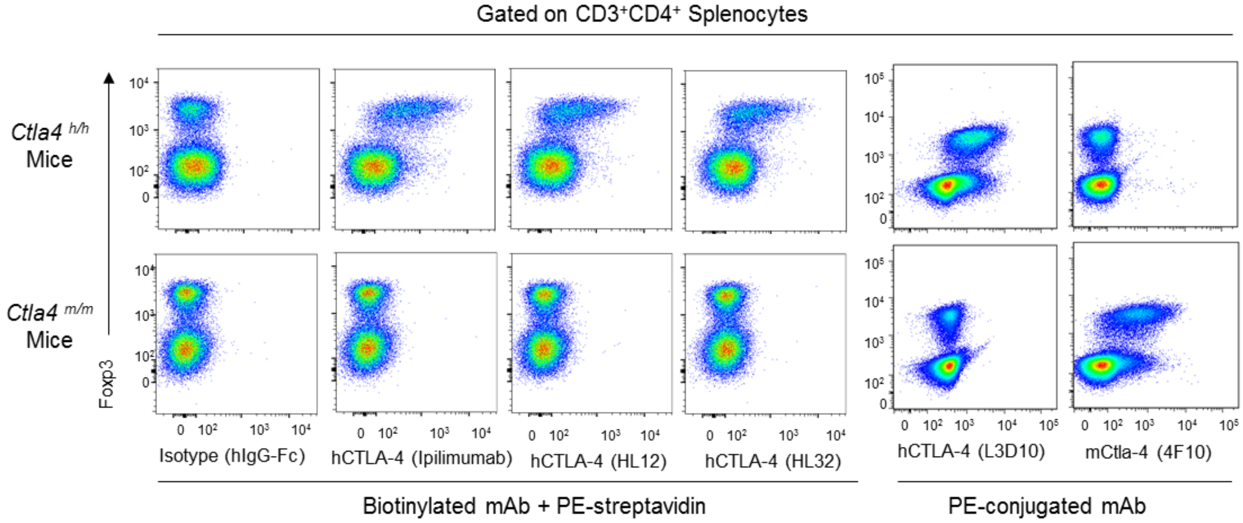

Supplementary information, Figure S5 L3D10, HL12, HL32 and Ipilimumab bind to human CTLA-4 but not mouse Ctla-4. Data shown are dot plots of intracellular staining of CTLA-4 among gated $CD3^+CD4^+$ cells, using spleen cells from *Ctla4*$^{h/h}$ (top) or *Ctla4*$^{m/m}$ (bottom) mice. Anti-mouse Ctla-4 mAb 4F10 (BD Biosciences) was used as control. Anti-CD3 (clone 145-2C11), CD4 (clone RM4-5), Foxp3 (clone FJK-16s) mAbs and Foxp3 staining buffer were purchased from eBioscience.
