# Supplementary material for: A reappraisal of CTLA-4 checkpoint blockade in cancer immunotherapy
Source: Cell Res. 2018 Feb 22;28(4):416–32. doi: 10.1038/s41422-018-0011-0 (PMC5939050; doi:10.1038/s41422-018-0011-0)
Supplement: Supplementary file 7 — Supplementary information, Figure S6 [file 41422_2018_11_MOESM7_ESM.pdf]

Figure S6

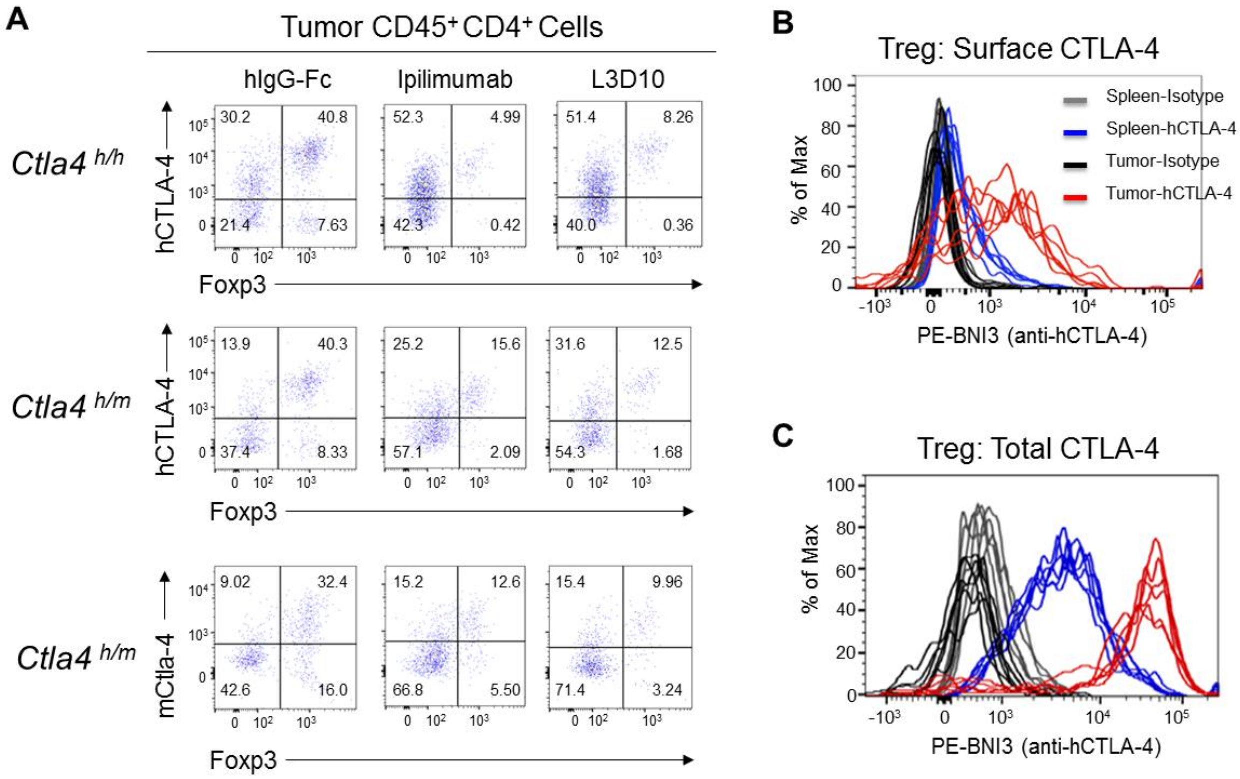

Supplementary information, Figure S6 CTLA-4 was preferentially highly expressed in tumor-infiltrated Tregs. **(A)**Tumor-derived Tregs had higher expression of CTLA-4 than Foxp3-negative CD4 T cells. As in Figure 7, MC38 tumor cells were injected into *Ctla4*$^{h/h}$ or *Ctla4*$^{h/m}$ mice and mice were treated with 100µg control hIgG-Fc or anti-CTLA-4 mAbs. Five days after the third antibody treatment, mice were sacrificed and tumor cells were subjected to flow cytometric analysis on human CTLA-4 or mouse Ctla-4 expression in tumor-infiltrated CD45$^+$CD4$^+$Foxp3$^+$ Tregs and CD45$^+$CD4$^+$Foxp3$^-$ T cells. Data represent the results from one of three independent experiments. **(B, C)** Tregs from tumor had higher expression of both surface CTLA-4 and total CTLA-4 than that from spleen. As in Figure 7, 14 days after MC38 tumor inoculation, *Ctla4*$^{h/h}$ mice were sacrificed for flow cytometric analysis on surface CTLA-4 (**B**) and total CTLA-4 (**C**) expression in spleen and tumor derived CD4$^+$Foxp3$^+$ Tregs. Each line of the histogram

plots indicates one individual mouse. n=6 mice and data shown represent the results from one of at least three independent experiments.
