# Supplementary material for: A reappraisal of CTLA-4 checkpoint blockade in cancer immunotherapy
Source: Cell Res. 2018 Feb 22;28(4):416–32. doi: 10.1038/s41422-018-0011-0 (PMC5939050; doi:10.1038/s41422-018-0011-0)
Supplement: Supplementary file 8 — Supplementary information, Figure S7 [file 41422_2018_11_MOESM8_ESM.pdf]

Figure S7

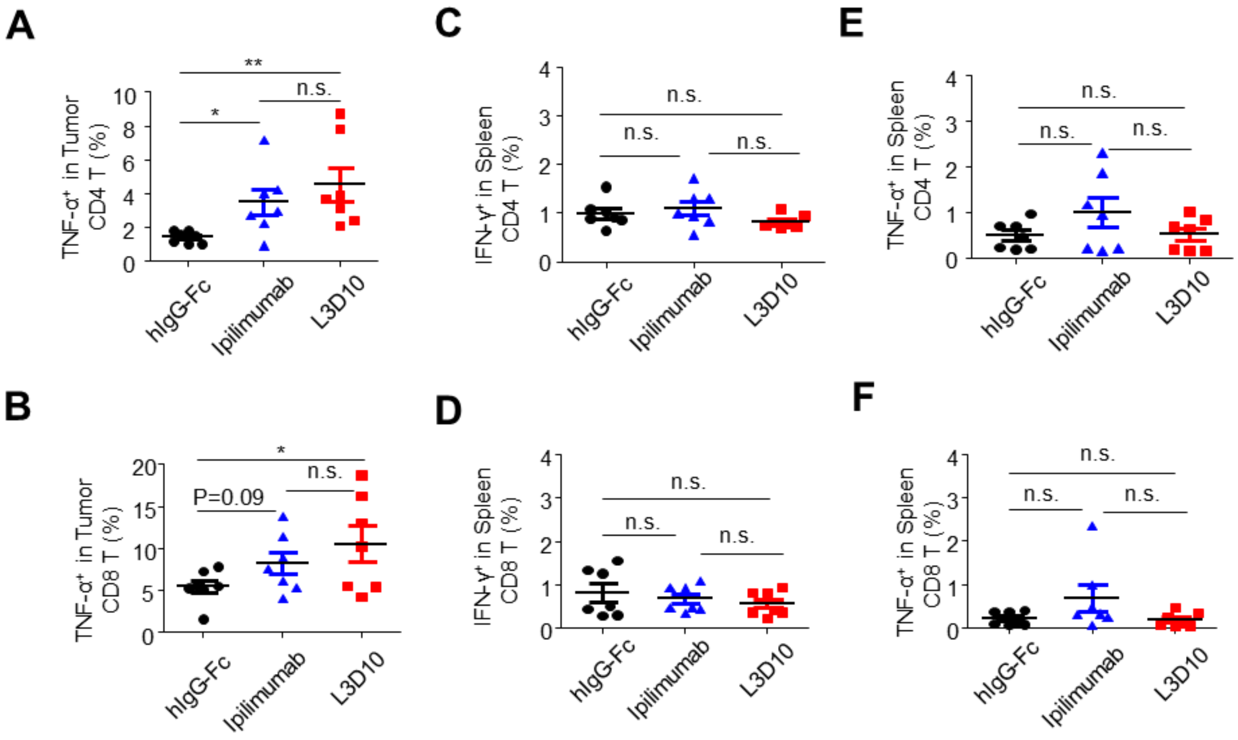

Supplementary information, Figure S7 Effects of anti-hCTLA-4 mAbs on IFNγ and TNFα production among spleen and tumor T cells. As in Figure 7A, MC38 tumor cells were injected into *Ctla4*$^{h/h}$ mice and mice were treated with 100µg control hIgG-Fc or anti-CTLA-4 mAbs. Three days after the third antibody treatment, mice were sacrificed to analyze the frequencies of IFNγ– and TNFα–expressing cells among CD4 (**A**, **C**, **E**) and CD8 (**B**, **D**, **F**) T cells in tumors (**A**, **B**) and spleens (**C**-**F**) from the treated mice. Summary data are from two experiments involving 7 mice per group.
