# Supplementary material for: A reappraisal of CTLA-4 checkpoint blockade in cancer immunotherapy
Source: Cell Res. 2018 Feb 22;28(4):416–32. doi: 10.1038/s41422-018-0011-0 (PMC5939050; doi:10.1038/s41422-018-0011-0)
Supplement: Supplementary file 9 — Supplementary information, Figure S8 [file 41422_2018_11_MOESM9_ESM.pdf]

Figure S8

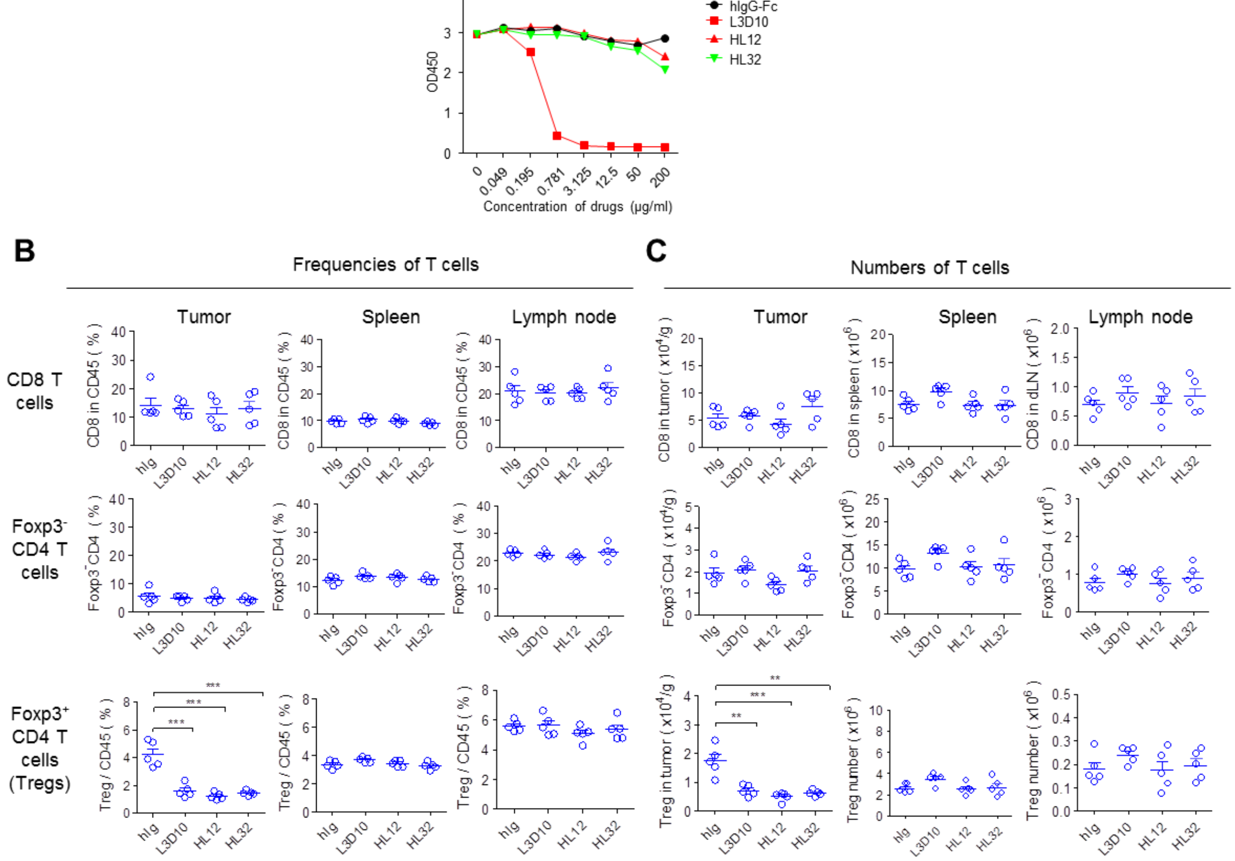

Supplementary information, Figure S8 Despite the inability to block CTLA-4-B7 interaction, HL12 and HL32 exhibit similar effects as L3D10 on abundance of T cell subpopulations in peripheral lymph organs and tumors. **(A)**The ability of HL12 and HL32 to block soluble B7 binding to immobilized CTLA-4-Fc was abrogated. hCTLA-4-Ig was immobilized at the concentration of 0.25μg/ml on 96-well ELISA plate. Biotinylated hB7-1-Fc was added at 0.25μg/ml along with giving doses of anti-CTLA-4 mAbs (L3D10, HL12 and HL32) or control hIgG-Fc. After washing, the plate-bound biotinylated hB7-1-Fc was detected with HRP-conjugated avidin. Data shown are means of triplicate optical density at 450 nm. Results are representative of 3 independent experiments. **(B, C)** L3D10, HL12 and HL32 preferentially eliminate tumor-infiltrated Tregs. As in Figures 8E-8G, the frequencies (**B**) and numbers (**C**) of CD8 T cells (top row), $CD4^+Foxp3^-$ T

cells (middle row) and $CD4^+Foxp3^+$ Tregs (bottom row) in tumor, spleen and tumor draining lymph node (dLN) were analyzed. Live $CD45^+$ leukocytes were initially gated to quantitate the frequencies of T cell subpopulations (T subset/$CD45^+$ cells X 100%) in various tissues, and the numbers of T cell subpopulations in tumors were normalized against tumor weight (gram). Mice were sacrificed one day after one injection of 100μg indicated drug. Data shown were pooled from 2 experiments. n=5 mice for each group.
