# Supplementary material for: A reappraisal of CTLA-4 checkpoint blockade in cancer immunotherapy
Source: Cell Res. 2018 Feb 22;28(4):416–32. doi: 10.1038/s41422-018-0011-0 (PMC5939050; doi:10.1038/s41422-018-0011-0)
Supplement: Supplementary file 10 — Supplementary information, Figure S9 [file 41422_2018_11_MOESM10_ESM.pdf]

Figure S9

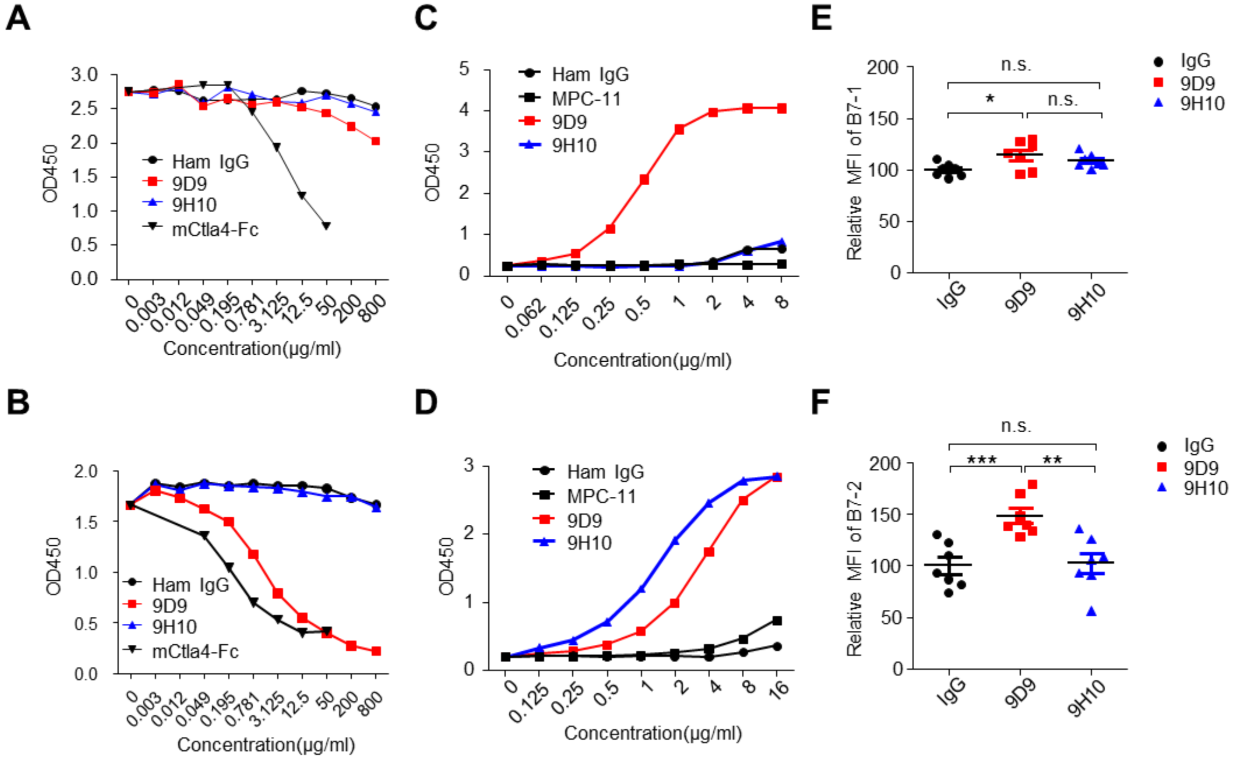

Supplementary information, Figure S9 Evaluation of blocking activities of commonly used anti-mouse Ctla-4 mAbs 9H10 and 9D9. **(A, B)** 9H10 does not block B7-CTLA-4 interaction if B7-1 (**A**) and B7-2 (**B**) are coated onto plates. Biotinylated mouse Ctla-4-Fc fusion protein were incubated with B7-coated plates in the presence of given concentration of control IgG or anti-mouse Ctla-4 mAb 9D9 and 9H10. Data shown are means of duplicated wells and are representative of two independent experiments. **(C, D)** 9D9 and 9H10 exhibit differential binding ability to soluble (**C**) and plate bound Ctla-4-Fc (**D**). MPC-11(mouse IgG2b) and Hamster IgG (Ham IgG) are isotype-matched control Ig proteins. Data shown are means of duplicated wells and are representative of at least two independent experiments. **(E, F)** Differential effect of anti-mouse Ctla-4 mAbs 9D9 and 9H10 on upregulating the levels of B7-1 (**E**) and B7-2 (**F**) on splenic CD11c^{high} DCs from WT (*Ctla4*$^{m/m}$) mice. At 24 hours after treatment with 500 μg

antibodies, mice were sacrificed and splenocytes were harvested for flow staining immediately. IgG group indicates mice receiving 500 μg of MPC-11 and 500μg of Ham IgG. The data (Mean ± S.E.M.) are summarized from 6 independent mice per group in two independent experiments involving 3 mice per group each. Statistical significance in e and f was determined using Student's *t* test. **P*<0.05, ***P*<0.01, ****P*<0.001. n.s., not significant.
