# Supplementary material for: A reappraisal of CTLA-4 checkpoint blockade in cancer immunotherapy
Source: Cell Res. 2018 Feb 22;28(4):416–32. doi: 10.1038/s41422-018-0011-0 (PMC5939050; doi:10.1038/s41422-018-0011-0)
Supplement: Supplementary file 11 — Supplementary information, Figure S10 [file 41422_2018_11_MOESM11_ESM.pdf]

Figure S10

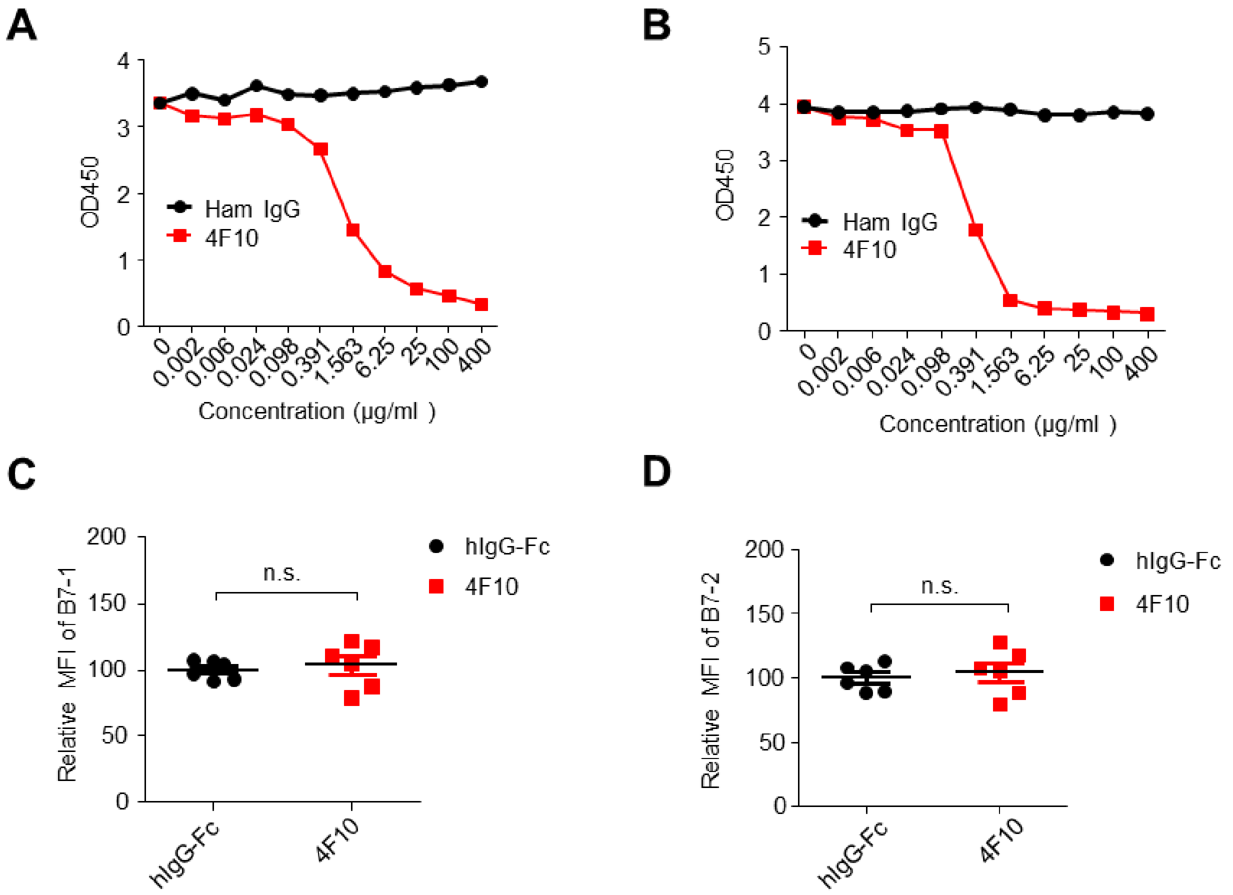

Supplementary information, Figure S10 Distinct *in vitro* and *in vivo* blocking activities of anti-mouse Ctla-4 mAb 4F10. **(A, B)** The effect of 4F10 on interaction of Ctla-4Fc to plate-coated B7-1 (**A**) or B7-2 (**B**). Biotinylated mouse Ctla-4-Fc fusion protein was incubated with B7-coated plates in the presence of given concentrations of control IgG or anti-mouse Ctla-4 mAb 4F10. The Ctla-4 binding was detected with HRP-conjugated avidin. Data shown are means of duplicates and are representative of two independent experiments. **(C, D)** Impact of 4F10 on B7-1 and B7-2 expression on CD11c^{high} dentritic cells. Spleen cells from WT (*Ctla4*$^{m/m}$) mice administrated i.p. with 500μg 4F10 or hIgG-Fc were analyzed for B7 levels by flow cytometry. Summary data (Mean ± S.E.M.) on

B7-1 (**C**) and B7-2 (**D**) levels are from 6 mice per group. The B7 levels in the control IgG-treated group are artificially defined as 100.
